# Effects of Multicomponent Digital Health Interventions on Multidimensional Physical Activity in Older Adults: Systematic Review, Meta-Analysis, and Meta-Regression of Randomized Controlled Trials

**DOI:** 10.2196/91338

**Published:** 2026-05-29

**Authors:** Jiayi Yao, Haozhe Wang, Shiguan Jia, Hao Chen, Junhao He, Juncheng Long, Shengxian Chen

**Affiliations:** 1 School of Physical Education, China University of Mining and Technology, No. 1 University Road, Xuzhou, Jiangsu, China; 2 School of Marxism, Jiangsu Normal University, Xuzhou, Jiangsu, China; 3 School of Physical Education and Training, Capital University of Physical Education and Sports, Beijing, China; 4 Department of Physical Education and Research, Beijing City University, No. 26 Yangzhen Section, Muyan Road, Yangzhen, Shunyi District, Beijing, 101309, China, 86 18600483371

**Keywords:** older adults, digital health interventions, physical activity, sedentary behavior, randomized controlled trials, meta-analysis, meta-regression

## Abstract

**Background:**

The comprehensive effects of multicomponent digital health interventions (DHIs) on multidimensional physical activity indicators and sedentary behavior (SB) remain controversial.

**Objective:**

This systematic review aimed to evaluate the impact of multicomponent DHIs on daily steps, moderate-to-vigorous physical activity (MVPA), light physical activity, total physical activity, and SB in older adults.

**Methods:**

PubMed, Web of Science, Embase, The Cochrane Library, and CINAHL were searched up to February 20, 2026. Randomized controlled trials concerning multicomponent DHIs for promoting exercise behavior in older adults were included. RoB 2.0 was used to evaluate study quality. Meta-analyses were performed using the Hartung-Knapp-Sidik-Jonkman random-effects model, and 95% prediction intervals (PIs) were calculated via Nagashima adjustment to evaluate effect dispersion. The GRADE (Grading of Recommendations, Assessment, Development, and Evaluation) system was used to evaluate evidence certainty.

**Results:**

A total of 26 randomized controlled trials (n=4129) were included. The results showed that multicomponent DHIs significantly improved daily steps (mean difference [MD] 822.8, 95% CI 198.3 to 1447.3 steps/d; 95% PI –1452.4 to 3098.0) and MVPA (MD 45.9, 95% CI 23.9 to 67.9 min/wk; 95% PI –9.4 to 101.2). However, the improvements in SB (MD –283.7, 95% CI –610.8 to 43.5 min/wk; 95% PI –984.5 to 417.1), total physical activity (MD 104.4; 95% CI –109.2 to 318.0 min/wk; 95% PI –444.4 to 653.2), and light physical activity (MD 39.3, 95% CI –96.2 to 174.7 min/wk; 95% PI –227.6 to 306.2) did not reach statistical significance. As some included studies combined digital tools with human support, the independent contribution of digital technology remains uncertain. PIs indicated a certain degree of dispersion across different clinical contexts. Subgroup analysis showed higher effect sizes for standalone wearables, human-assisted interventions, and populations with chronic disease risks. Meta-regression showed that effect sizes remained stable across different ages and durations. The trim-and-fill method confirmed the robustness of MVPA results. GRADE assessment indicated “moderate” certainty for MVPA and “low” for daily steps and other indicators.

**Conclusions:**

This systematic review suggests that multicomponent DHIs may serve as an effective means for enhancing daily steps and MVPA in older adults. The innovation lies in evaluating the true effect distribution of multicomponent DHIs through Hartung-Knapp-Sidik-Jonkman random-effects models and Nagashima PIs. Compared with previous studies, this review identified the impact of population characteristics and control group differences on effect estimates using PI and subgroup models, confirming that advanced age did not significantly diminish the good adaptability of older adults to DHIs. Evidence limitations include high heterogeneity, lack of long-term follow-up, and differences between objective and subjective measurement tools. In practice, priority should be given to hardware carriers with simplified interaction and integrated human support, with tailored strategies developed for different risk subgroups.

## Introduction

With the accelerating process of global population aging, maintaining the physical function and independent living capacity of the older population has become a core element of public health strategies [1,2]. Physical activity (PA) is widely recognized as a key protective factor for preventing chronic noncommunicable diseases, maintaining muscle mass, and improving mental health [3,4]. However, there is a significant imbalance in participation across different intensities of PA among older adults. Most older individuals struggle to reach recommended levels of moderate-to-vigorous physical activity (MVPA), which directly limits the maintenance of cardiorespiratory fitness and metabolic health [5]. Meanwhile, sedentary behavior (SB) accounts for 65% to 80% of the waking hours of older adults; this pattern of prolonged physical inactivity is closely associated with an increased risk of all-cause mortality [6]. Although light physical activity (LPA) is considered an effective alternative for reducing SB and improving health outcomes in older adults, systematic attention toward LPA remains relatively scarce in existing intervention studies and guideline recommendations [7].

Multicomponent digital health interventions (DHI) provide important means for promoting improvements in exercise behavior among older adults by integrating behavior change techniques such as goal setting, self-monitoring, automated feedback, and social support [8,9]. Currently, intervention formats encompass various digital technology carriers, including mobile apps, standalone wearable electronic devices, and web-based interactive platforms, with applications expanding from general community-dwelling healthy older adults to specific groups with health risks [10,11]. Furthermore, as intervention programs often comprise multidimensional components, they exhibit significant heterogeneity in delivery agents [12]. One category is “technology-driven,” where automated algorithms (such as fully automated app programs) serve as the primary interactive entity to achieve real-time goal adjustment and feedback. The other category is “technology-assisted,” where digital technology primarily serves as an auxiliary monitoring tool, while the core behavior change support still relies on human involvement (such as health care professionals or coaches). Compared with traditional face-to-face intervention programs, multicomponent digital interventions demonstrate significant advantages in reducing resource consumption, increasing accessibility, and enhancing the convenience of long-term management [13]. Additionally, an increasing number of digital programs are being designed based on established theoretical frameworks, such as Social Cognitive Theory and the Health Action Process Approach, aiming to enhance exercise adherence in older adults through precise monitoring data and personalized interactive experiences [14].

Although several meta-analyses have confirmed the positive effects of multicomponent DHIs on increasing daily steps and MVPA, existing reviews still possess various limitations [15]. Currently, the composition of the participant populations included in most studies is extremely complex, often mixing generally healthy individuals, those at risk for chronic diseases, and various patients already in clinical treatment stages. This leads to ambiguity regarding the guiding significance of research conclusions for healthy community-dwelling older adults or specific at-risk populations [16]. Such population heterogeneity limits a deep understanding of intervention suitability, making it difficult to clarify the precise efficacy of multicomponent digital technology in populations transitioning from a healthy state to a diseased state. Simultaneously, existing reviews provide an imbalanced assessment of different PA intensity indicators. In particular, regarding the effects on reducing SB and increasing LPA, conclusions across different studies vary significantly and lack a systematic summary [17]. Due to the diversity of technical carriers and interaction methods, existing research rarely directly compares the advantages and disadvantages of standalone wearable devices vs mobile apps and web platforms in terms of intervention effects [18]. Furthermore, most studies primarily report the average pooled effect after intervention, failing to fully explain the sources of significant interstudy heterogeneity. Although participants’ age, sex ratio, and intervention duration are considered key moderating variables affecting intervention outcomes, in-depth meta-regression analyses targeting these specific covariates remain scarce in this field. Consequently, the internal laws governing how intervention efficacy evolves or varies by participant characteristics have not been fully revealed [19]. The application of tools to assess the certainty of pooled evidence remains insufficiently widespread in existing reviews, which also limits the applied value of research conclusions.

Therefore, this systematic review aims to comprehensively examine the multidimensional effects of multicomponent DHIs on daily steps, MVPA, SB, LPA, and total physical activity (TPA) in older adults through systematic evaluation and meta-analysis, thereby providing a complete picture of changes in exercise behavior among older adults. This systematic review not only focuses on the average pooled effect generated by interventions but also evaluates the distribution and generalizability of intervention effects across different research contexts through prediction intervals (PIs). Meanwhile, this systematic review will compare differences in intervention efficacy between standalone wearable devices and models involving mobile apps or web platforms. It will also use meta-regression analysis to quantitatively explore the moderating effects of age, intervention duration, and prompt frequency on effect sizes, thereby assessing the stability of multicomponent DHI effects across different older populations and implementation schemes. Finally, this systematic review will rigorously evaluate the certainty of evidence for each outcome indicator in conjunction with the GRADE (Grading of Recommendations, Assessment, Development, and Evaluation) system, aiming to provide rigorous evidence-based support for developing precise digital health management strategies for older adults.

## Methods

### Study Design

This systematic review protocol was registered with PROSPERO (CRD420261323151). After the research team conducted the initial presearch in December 2025, it was found that the originally designed inclusion or exclusion criteria and intervention definitions required refined adjustments based on preliminary evidence. To ensure high consistency between the registered protocol and the final implementation plan, the team updated and resubmitted the registration application in early 2026 after perfecting the protocol. Although it constitutes retrospective registration in terms of procedure due to the presearch, the final implementation plan, outcome measure definitions, and statistical analysis plan of this systematic review are entirely consistent with the design initially submitted to the platform, with no protocol deviations occurring. The implementation and reporting of this systematic review strictly follow the PRISMA (Preferred Reporting Items for Systematic Reviews and Meta-Analyses) 2020 statement [20], and the PRISMA-S (Preferred Reporting Items for Systematic Reviews and Meta-Analyses literature search extension) guideline [21] was adopted for the reporting of the search strategy to ensure the transparency and reproducibility of the search process.

### Search Strategy

The search process for this systematic review strictly follows the PRISMA-S extension statement. A systematic and comprehensive literature search was conducted on February 20, 2026, across the PubMed (via NCBI), Web of Science Core Collection (via Clarivate), The Cochrane Library (via Wiley), Embase (via Elsevier), and CINAHL (via EBSCOhost) databases, with the search timeframe spanning from the inception of each database to the date of the search. The search scheme was constructed based on 4 core dimensions: population, intervention, outcome, and study design, using Boolean logic operations combining subject headings (eg, MeSH [Medical Subject Headings], Emtree, and CINAHL Subject Headings) and free-text terms. To ensure search depth and precision, differentiated adaptations were made according to the search characteristics of each database. For example, expanded subject heading searches were combined with TIAB (title and abstract) field limitations in PubMed and Embase; the TS (topic) field was used for exact term matching in Web of Science; the NEXT proximity operator was applied in The Cochrane Library; and the XB (exact bound) qualifier was introduced in CINAHL to enhance the accuracy of subject heading matching. Specific terms encompassed “older adults” (“aged,” “older adults,” “elderly,” and “aging”), “digital health interventions” (“telemedicine,” “mobile app,” “wearable devices,” “mHealth,” and “eHealth”), and “physical activity indicators” (“PA,” “exercise,” “daily steps,” “MVPA,” and “SB”), with randomized controlled trial (RCT) filters applied for precise screening. Additionally, this systematic review identified extra records by manually tracking the reference lists of included studies (backward citation searching) and attempted to contact original authors via email to obtain unpublished raw data or missing information. No additional searches were performed for manually browsed online resources, conference proceedings, or specific manufacturers; meanwhile, the search strategy was developed and proofread internally by the research team without undergoing an external peer review process. Given that this systematic review design was limited to RCTs, this systematic review did not conduct specific searches of gray literature or preprint databases. Complete search steps and Boolean logic formulas for each database are detailed in Multimedia Appendix 1.

### Inclusion and Exclusion Criteria

The literature screening process strictly followed the PICOS principles: participants (P) included older adults with a mean age of ≥60 years, encompassing community-dwelling healthy individuals as well as specific populations with frailty, fall risks, or chronic disease risks; interventions (I) were defined as programs aimed at promoting PA that used multicomponent digital health technologies (eg, mobile apps, interactive web platforms, and smart wearable devices) as the core interactive carrier, with interventions categorized based on whether the digital technology served as the primary automated interactive entity or as an auxiliary tool for human-supported interventions; comparisons (C) were set as groups receiving usual care, basic health monitoring, pure health education, or those on a waitlist; outcomes (O) were required to include at least one of the following: daily steps, weekly MVPA, SB, LPA, or TPA volume; and study type (S) was limited to peer-reviewed, published RCTs, including randomized pilot trials and feasibility studies with clear randomization designs. Exclusion criteria were defined as (1) studies with nonrandomized controlled designs, case reports, or reviews; (2) research consisting only of study protocols or conference abstracts that lacked complete empirical results; (3) literature where original valid data could not be obtained or converted, and attempts to contact the authors were unsuccessful; and (4) intervention studies where digital technology served only as an auxiliary recording and measurement tool and did not involve any behavior change feedback or interactive functions.

### Data Collection Process and Data Items

Data extraction was performed independently by two researchers (JY and HW), referencing the Cochrane Handbook guidelines to record information. The extracted content encompassed basic study information, participant characteristics (age, sex, and health status), outcome indicators (daily steps, MVPA, LPA, TPA, and SB), and key details of the multicomponent digital interventions, such as technical carriers, theoretical frameworks, core behavior change techniques, and the level of human interaction. Any discrepancies during the extraction process were resolved through discussion or by consulting a third independent researcher (SC).

### Choice of Effect Measures and Handling of Missing Data

During the quantitative data extraction process, this systematic review followed a specific hierarchy of priorities: preference was given to effect estimates adjusted by models such as analysis of covariance; if unavailable, change scores from baseline to post intervention were calculated; if neither of the former was obtainable, postintervention means and SDs were used. For missing SDs during the calculation of change scores, this systematic review referred to methods recommended in the Cochrane Handbook, setting the correlation coefficient at 0.5 for estimation [22]; in cases of missing data or unclear reporting, the original authors were contacted via email to obtain supplementary information.

### Assessment of Risk of Bias

The quality of the included studies was evaluated using the Cochrane RoB 2.0 tool [23]. The assessment covered bias arising from the randomization process, bias due to deviations from intended interventions, bias due to missing outcome data, bias in the measurement of the outcome, and bias in the selection of the reported results. Each domain, as well as the overall risk, was rated as low risk, some concerns, or high risk. SJ and HC performed the evaluations independently, resolving disagreements through discussion.

### Evidence Certainty Assessment

This systematic review used the GRADE system to assess the certainty of evidence for pooled outcome indicators, comprehensively considering the risk of bias, inconsistency, indirectness, imprecision, and publication bias. In evaluating inconsistency, the initial heterogeneity encompassing all relevant studies served as the basis for downgrading decisions. The quality of evidence was ultimately classified into four levels: high, moderate, low, or very low [24].

### Data Analysis

All statistical analyses for this systematic review were performed using R (R Foundation) software via the *metafor* and *forestplot* packages. Although individual studies varied in measurement tools and intervention protocols, given that daily steps, MVPA, LPA, TPA, and SB all possess unified physical units and clear clinical interpretative value, this systematic review used the mean difference (MD) and its 95% CI values for effect size pooling. To ensure the rigor of MD pooling, all data were converted to standard dimensions before analysis. Furthermore, this systematic review conducted sensitivity analyses using the standardized mean difference (SMD) to evaluate the impact of measurement tool diversity on the robustness of the pooled results. Considering the potential clinical and methodological heterogeneity among studies, this systematic review consistently adopted a random-effects model for effect size pooling. For multiarm studies involving multiple intervention groups compared against a single control group, to avoid overstating the evidence due to double-counting of samples, this systematic review followed Cochrane Handbook recommendations by equally dividing the control group sample size among the comparison groups while keeping the means and SDs constant. Additionally, to reduce the false positive rate in the random-effects model and provide more robust CIs, the Hartung-Knapp-Sidik-Jonkman (HKSJ) method was used to adjust all pooled effect sizes and *P* value calculations [25].

Statistical heterogeneity was quantitatively evaluated using the Q-test and the *I*^2^ index. Notably, given that *I*^2^ only reflects the proportion of observed variation caused by true differences in effect, to further quantify the clinical significance of heterogeneity in real-world settings, 95% PIs were calculated when the number of included studies was ≥3. Unlike the 95% CI, which reflects the precision of the average effect, the 95% PI quantifies the range of variation of the true effect across different populations or settings [26]. For significant outcome indicators, sensitivity analyses were conducted using the “leave-one-out” method to assess the robustness of pooled results and identify sources of heterogeneity. This systematic review preset subgroup classification criteria across five dimensions to address sources of heterogeneity. First, intervention duration was divided into short-term and medium-to-long-term using a 12-week threshold; second, population types were categorized into those with specific chronic disease risks and generally healthy older adults; third, control group settings were divided into active controls, providing routine exercise guidance or time-matched education, and passive controls, maintaining the status quo or joining a waitlist; fourth, technical carriers were categorized into standalone wearable devices and mobile apps; finally, delivery agent models were classified based on the nature of human-computer interaction into technology-driven and technology-mediated types, with the former providing automated feedback via algorithms or sensors without real-time human intervention, and the latter using digital platforms with remote guidance or personalized feedback provided by professionals [27]. Simultaneously, random-effects meta-regression analysis was performed on primary outcome indicators using age, intervention duration, female proportion, and prompt frequency as covariates [28]. This systematic review assessed the presence of small-study effects by observing funnel plot symmetry combined with Egger linear regression test (when the number of included studies k was >10) [29]. It should be noted that funnel plot asymmetry may stem from various factors such as publication bias, differences in study quality, or heterogeneity. If significant asymmetry was detected, this systematic review used the trim-and-fill method to adjust the pooled effect size, further evaluating the impact of potential bias on the robustness of the conclusions.

## Results

### Literature Search and Study Selection

**Figure 1.**
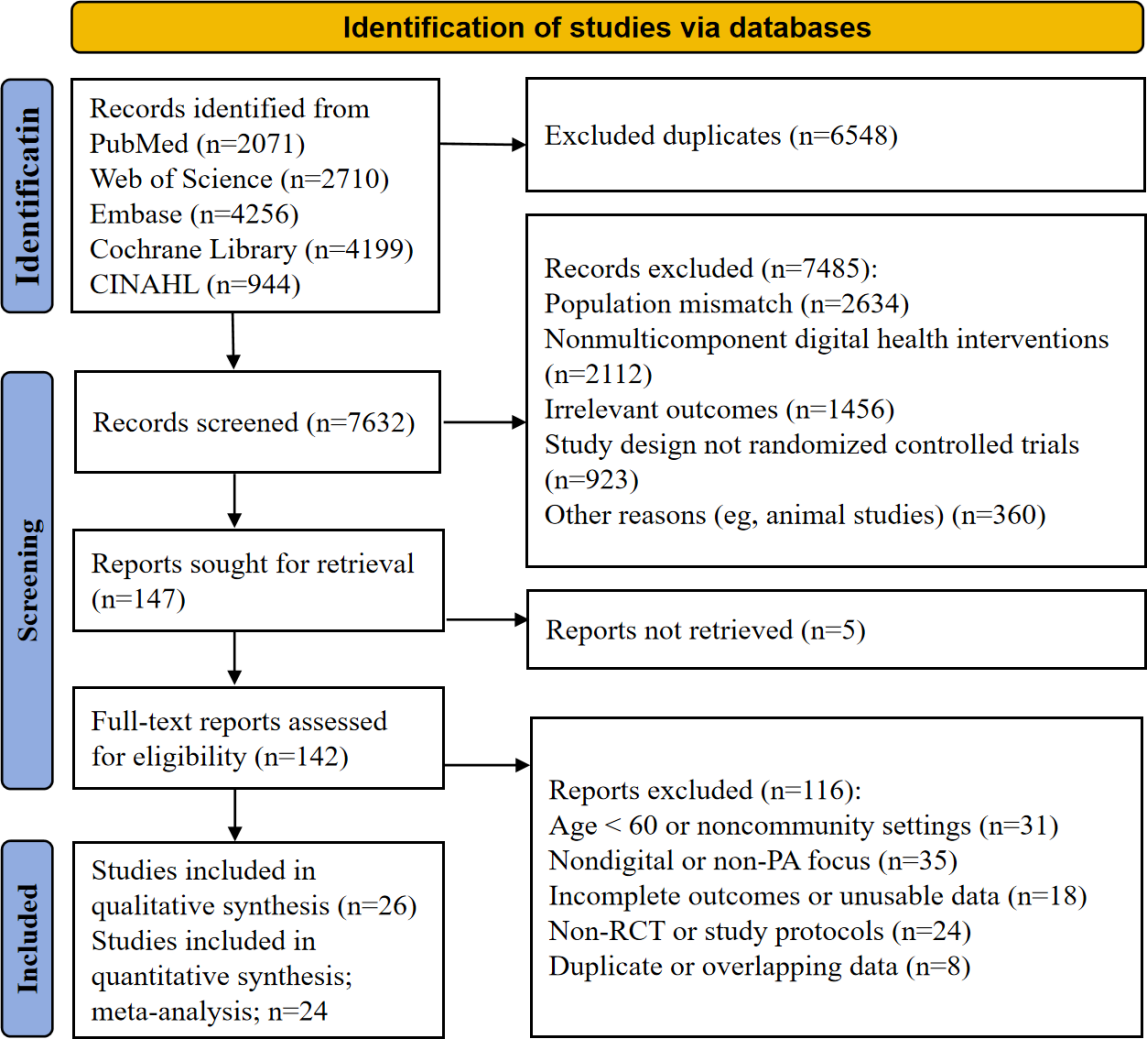
PRISMA 2020 flowchart of literature search and selection for multicomponent digital health interventions to improve PA levels in older adults. PA: physical activity; PRISMA: Preferred Reporting Items for Systematic Reviews and Meta-Analyses; RCT: randomized controlled trial.

The search process for this systematic review strictly followed the PRISMA-S extension criteria (Figure 1). Through systematic searches of the PubMed (via NCBI), Web of Science Core Collection (via Clarivate), The Cochrane Library (via Wiley), Embase (via Elsevier), and CINAHL (via EBSCOhost) databases, a total of 14,180 original records were obtained. After using the automated tool EndNote (Clarivate) to remove 6548 duplicate records, a preliminary screening of titles and abstracts was conducted for the remaining 7632 records, resulting in the exclusion of 7485 irrelevant documents. The primary reasons for excluding these records included a clear deviation in the research topic, nonoriginal research types, or a distinct mismatch with the target population. During the full-text retrieval of the remaining 147 reports, 5 reports could not be obtained in full. Subsequently, researchers performed a rigorous eligibility assessment on 142 full-text papers, excluding a total of 116 documents for specific reasons: participants in acute rehabilitation phases or noncommunity or rural-dwelling groups (n=31); lack of digital carriers or non-PA interventions (n=35); missing outcome measures or unusable data (n=18); non-RCTs or study protocols only (n=24); and duplicate publications or studies with overlapping data (n=8). Following this screening process, 26 studies were ultimately included in the systematic review, of which 24 studies were included in the meta-analysis due to having sufficient quantitative data.

### Description of Included Study Characteristics

The 26 RCTs ultimately included in this systematic review were published between 2009 and 2025 [30-55], encompassing various design types such as cluster RCTs, crossover RCTs, and randomized pilot studies. These RCTs involved a total of 4129 participants, with research areas spanning Australia [35,36,43,45,49], the United States [37,40,42,44], China [33,38,41,46,48,51], the United Kingdom [30,54], Japan [47,52], and other countries [31,32,34,39,50,53,55]. Intervention durations varied significantly, ranging from 4 weeks [38] to 12 months of continuous intervention or follow-up [39,43,50,54], with short-to-medium-term interventions of 12 and 24 weeks being the most common. The mean age of participants was over 60 years, ranging from 60.0 [37] to 77.4 [35] years. Sample sizes per study ranged from 13 [32] to 605 [53], with a notable female predominance. Beyond healthy community-dwelling older adults, this systematic review populations widely involved groups at high risk for cardiovascular disease [44], impaired glucose tolerance [54], frailty or prefrailty [33,38,46], cognitive decline risk [51], fall risk [30], and overweight or obese sedentary high-risk groups [37,41,42].

In terms of intervention measures and technology implementation, digital health carriers showed a diverse trend, primarily including mobile apps [31,33,38,41,48,51,52], wearable electronic devices [32,36,37,42-46,49,53], and web-based interactive platforms [34,35,39,40,47,48,53]. Most intervention protocols were based on established behavior change theories, such as Social Cognitive Theory [32,42-45,47,48], the Health Action Process Approach [34,48], the Theory of Planned Behavior [45], and the Behavior Change Wheel [33]. Core intervention components typically included goal setting, self-monitoring, automated feedback, and social support. Regarding control group settings and outcome evaluation, control groups were mostly set to receive standard health education [31-33,35,48,49,55], usual care [30,36,37,41,46,54], email-based information [47], or waitlist status [38,40,45,53]. Outcomes were primarily measured using objective tools, such as ActiGraph GT9X accelerometers [45], ActivPAL sensors (PAL Technologies Ltd) [42,46], or smart pedometers [30,48-50,54]. Regarding data sources for effect size calculation among the 24 studies [32-55] included in the quantitative synthesis, 16 (66.7%) studies [32-38,40,42-47,49,53] used change scores from baseline; 6 (25%) studies [41,50-52,54,55] converted medians and IQRs into means and SDs; and the remaining 2 (8.3%) studies [39,48] used unadjusted postintervention data. Two studies, Audsley et al (2020) [30] and Saçıkara and Cingil (2024) [31], were excluded from the quantitative synthesis because they only reported the magnitude of change or qualitative trends in PA without providing the raw mean and SD values required for effect size calculation (details are shown in Table 1).

**Table 1.** Characteristics of the 26 included RCTs^a^ on multicomponent digital health interventions for improving physical activity levels in older adults.

Study (author/year)	Int/FU^b^ duration	Sample size (IG/CG^c^ initial/completed)	Age (years), mean (SD)	Sex (% female)	Health status/population	Digital platform and theoretical framework	Core BCTs^d^	Control setting	Outcomes and tools
Yates et al, 2009 [54]	12 months / 12 months	33+31/34 (initial) 29+28/26 (complete)	65 (8)	34	IGT^e^	Pedometer; diabetes prevention or self-efficacy theory	Education, personalized goals or steps, diary, action planning	Usual care (brief)	Steps; obj^f^: NL-800 pedometer
Bickmore et al, 2013 [50]	12 months / 12 months	263 (initial)	71.3	61	Sedentary urban older adults	Tablet+virtual agent; ECA^g^ theory	Daily automated dialogue, daily goal setting, automated coaching	Pedometer group	Steps; pedometer (obj)
Ashe et al, 2015 [32]	6 months / 6 months	13/13 (initial) 12/8 (complete)	64.1 (4.5)	100	Sedentary older women	Fitbit One (Fitbit Inc); SCT^h^/social ecological model	Goal setting, self-monitoring, feedback, social support, planning	Health education	MVPA^i^; ActiGraph (obj; ActiGraph LLC)
Cadmus-Bertram et al, 2015 [37]	16 weeks / N/A	25/26 (initial) 24/25 (complete)	60.0 (5.2)	100	Overweight/obese sedentary older women	Fitbit One+web; CALO-RE^j^ framework	Self-monitoring, goal setting, behavioral feedback, phone counseling	Usual care	MVPA; ActiGraph (obj)
Mouton and Cloes, 2015 [39]	3 months / 12 months	52+3/50 (initial) 149 completed	65.0 (6.0)	64	Insufficiently active older adults	Web platform+email; TTM^k^/ecological model	SMART^l^ goals, PA^m^ diary, feedback, forum social support	No intervention	Total PA; IPAQ-S^n^ (Sub^o^)
Muellmann et al, 2019 [53]	10 weeks / 10 weeks	104+107/107 (initial) 85+93/90 (complete)	68.6 (5.5)	64	Insufficiently active older adults	Web platform+Fitbit (Fitbit Inc); self-regulation theory	Goal setting, action or coping planning, feedback, self-monitoring	Waitlist control	Steps/MVPA/SB^p^; ActiGraph (obj)
Oliveira et al, 2019 [49]	6 months / 6 months	64/67 (initial) 54/57 (complete)	71.1 (7.1)	65	General older adults	Fitbit/pedometer; BCTs	GAS^q^, telephone coaching, feedback	Standard health education	MVPA/steps/LPA^r^/total PA; ActiGraph (obj)
Roberts et al, 2019 [44]	20 weeks / 20 weeks	20/20 (initial) 18/19 (complete)	72.0 (7.4)	60	CVD^s^ risk /high sedentary behavior	Fitbit Zip (Fitbit Inc); cognitive behavioral theory	Monitoring feedback, weekly SMS, goal setting	Structured exercise counseling	Steps/SB; ActiGraph (obj)
Van Dyck et al, 2019 [34]	5 weeks / 3 months	38/34 (initial) 34/31 (complete)	70.9 (4.8)	49	General older adults	MyPlan 2.0 Web (Ghent University); self-regulation or HAPA theory	Goal setting, action planning, coping planning, feedback	No intervention	Total PA/MVPA; IPAQ^t^ (sub)
Audsley et al, 2020 [30]	6 months / 6 months	20/30 (initial) 20/25 (complete)	IG: 76.9 (7.0)/CG: 73.8 (6.4)	80	Older adults at risk of falling	Pedometer+telephone; MI^ac^	MI counseling, PA diary, goal setting, action planning	Usual care	MVPA; Phone-FITT (sub; McGill University)
Kwan et al, 2020 [51]	12 weeks / 13 weeks	16/17 (initial) 15/15 (complete)	71.0 (9.0)^v^	85	Risk of cognitive decline	App+WhatsApp (WhatsApp LLC); persuasive technology	Personalized goals, monitoring, electronic reminders, video feedback	Routine behavior intervention	Steps/MVPA/total PA; ActiGraph (obj)
Rosenberg et al, 2020 [42]	12 weeks / 12 weeks	29/31 (initial) 29/25 (complete)	68.4 (4.9)	68	Overweight/obese sedentary older adults	Jawbone handband (Jawbone Inc); SCT/habit formation	Telephone coaching, goal setting, environment reminders, self-monitoring	Healthy life handbook	Steps/total PA; activPAL (obj; PAL Technologies Ltd)
Brickwood et al, 2021 [36]	12 weeks / 12 months	37+38/42 (initial) 24+25/26 (complete)	72.4 (6.1)	64	Risk of chronic diseases	Jawbone UP24 app (Jawbone Inc); SCT theory	Goal setting, app or SMS feedback, self-monitoring	Usual care	Steps; activPAL (obj)
Delbaere et al, 2021 [35]	24 months / 24 months	254/249 (initial) 201/212 (complete)	77.4 (5.4)	67	General older adults	Standing Tall app (NeuRA^w^); user-centered design/BCTs	Customized exercises, goal setting, feedback, educational manual	Electronic health education	LPA; MoveMonitor (obj; McRoberts)
Lin et al, 2025 [33]	14 weeks / 3 months	22/18 (initial) 22/18 (complete)	IG: 72.1 (3.7), CG: 80.4 (6.8)	85	Frail/prefrail older adults	Fitbit+app; BCTs	Goal setting, incentive badges, monitoring, action cues	Training+ lectures	Steps/MVPA; ActiGraph (obj)
Zhou et al, 2021 [41]	12 weeks / N/A	40/41 (initial) 34/34 (complete)	67.4 (5.1)	66	Overweight/obese older adults	App+smartband; BCTs	Goal setting, diet or PA monitoring, feedback, WeChat social support	Pedometer hand manual	Steps/MVPA; smartband (obj)
Alley et al, 2022 [45]	12 weeks / 24 weeks	78+96/69 (initial) 56+59/51 (complete)	69.34 (4.32)	78.6	Insufficiently active older adults	Web-based+Fitbit; TPB^x^/SCT	6 tailored suggestion modules, action planning, exercise video library	Waitlist control	MVPA/SB/Total PA/Steps; ActiGraph
Cai et al, 2022 [48]	3 months / N/A	36/36 (initial) 34/30 (complete)	66.9 (4.2)	64	Rural older adults	WeChat (Tencent Holdings Ltd)+pedometer; SCT/HAPA^y^ model	Peer support, planning, microexperience sharing	Standard health education	Steps; pedometer (obj)
Pischke et al, 2022 [40]	9 months / 9 months	242 (initial) 160 (complete)	68.7 (5.4)	62	Insufficiently active older adults	Web/app+Fitbit; self-regulation theory	Monitoring, goals, feedback, small group coaching, social support	Waitlist control	MVPA/SB; ActiGraph (obj)
Recio-Rodríguez et al, 2022 [55]	3 months / 3 months	81/79 (initial)	70.8 (4.0)	61.3	General older adults	App+smartband; behavior change theory	Lifestyle counseling, self-monitoring, dietary logs, automated feedback	Short behavior intervention	Steps; accelerometer/smartband
Kawaguchi et al, 2024 [52]	12 weeks / 12 weeks	87/94 (initial) 80/85 (complete)	IG: 69.9 (6.0), CG: 70.1 (6.7)	53	General older adults	ESP app (LINE Corporation)+LINE (LINE Corporation); BCTs	GPS trajectory monitoring, rank-based rewards, educational columns	Basic monitoring (Google Fit [Google LLC])	Steps; Google Fit (obj)
Oliveira et al, 2024 [43]	12 months / 12 months	290/315 (initial) 258/252 (complete)	74.0 (8.0)	70	General older adults	Fitbit+telephone; SDT theory	Risk assessment, goal setting, coaching, problem solving	Nutritional coaching	Steps; ActiGraph (obj)
Saçıkara and Cingil, 2024 [31]	8 weeks / 6 months	33/33 (initial) 33/27 (complete)	66.3 (4.2)	50	General older adults	Google Fit app; health promotion model	Health education, self-monitoring, small-group social interaction, rewards	No intervention	Total PA; scale (sub)
Uemura et al, 2024 [47]	12 weeks / 36 weeks	15/14 (initial) 14/12 (complete)	IG: 73.9 (3.9)/CG: 69.4 (3.2)	50	General older adults	Zoom (Zoom Video Communications, Inc) meeting; self-directed/SCT	Exploratory tasks, small group discussion, goals, monitoring	Mail information push	Total PA/MVPA/LPA; ActiGraph
Lee et al, 2025 [38]	4 weeks / 3 months	19/19 (initial) 16/18 (complete)	71.8 (9.34)	71	Frail/prefrail older adults	Rehab-Fit app (Fujitsu Limited); Self-efficacy theory	Educational workshops, app guidance, video/audio feedback	Waitlist control	MVPA; ActiGraph (obj)
Li et al, 2025 [33]	6 months / 6 months	67/67 (initial) 52/54 (complete)	69.3 (5.1)	75	Prefrail older adults	mHealth app; LIFE^z^ model/BCW^aa^ theory	Personalized plans, video guidance, remote monitoring, feedback	Health education	MVPA/SB/LPA; ActiGraph (obj)

a RCT: randomized controlled trial.

b Int/FU: intervention/follow-up.

c IG/CG: intervention group/control group.

d BCT: behavior change technique.

e IGT: impaired glucose tolerance.

f Obj: objective measurement.

g ECA: embodied conversational agent.

h SCT: Social Cognitive Theory.

i MVPA: moderate-to-vigorous physical activity.

j CALO-RE: Coventry, Aberdeen, and London—Refined.

k TTM: transtheoretical model.

l SMART: Sequential Multiple Assignment Randomized Trial.

m PA: physical activity.

n IPAQ-S: International Physical Activity Questionnaire-Short Form.

o Sub: subjective or self-reported measurement frameworks.

p SB: sedentary behavior.

q GAS: goal attainment scaling.

r LPA: light physical activity.

s CVD: cardiovascular disease.

t IPAQ: International Physical Activity Questionnaire.

u MI: motivational interviewing.

v Median (IQR).

w NeuRA: Standing Tall App (Neuroscience Research Australia.

x TPB: Theory of Planned Behavior.

y HAPA: health action process approach.

z LIFE: Lifestyle Interventions and Independence for Elders.

aa BCW: behavior change wheel.

### Risk-of-Bias Assessment

**Figure 2.**
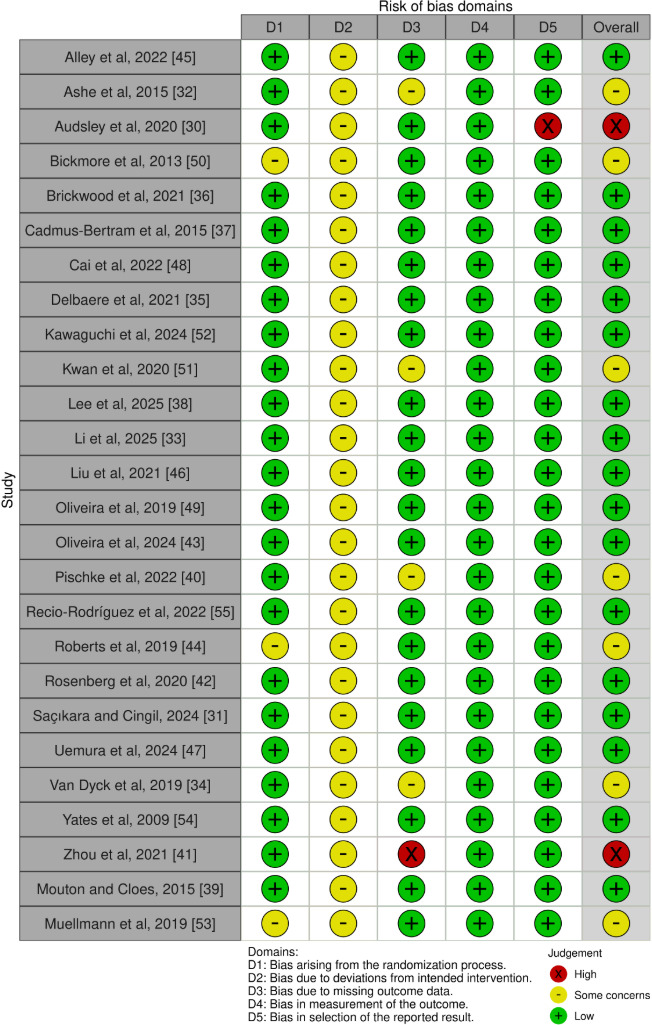
Risk of bias summary for the 26 included RCTs evaluating multicomponent digital health interventions for older adults. RCT: randomized controlled trial [30-55].

**Figure 3.**
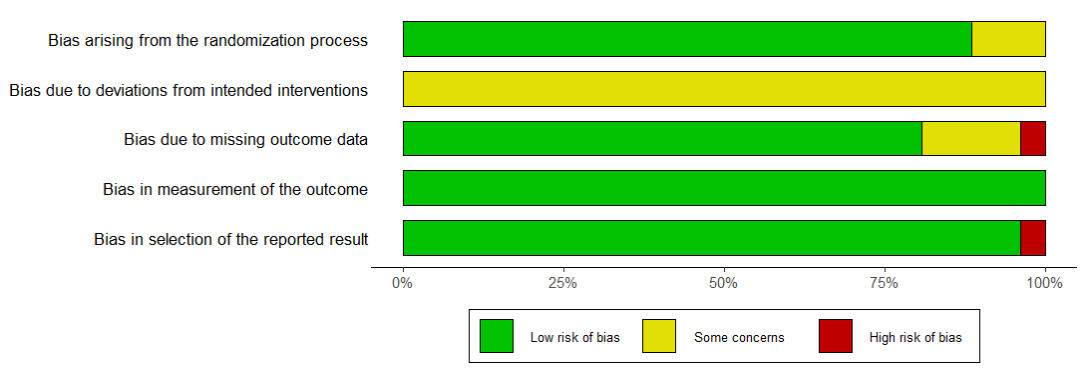
Risk-of-bias graph presented as cumulative percentages across the 26 included RCTs on multicomponent digital health interventions for older adults. RCT: randomized controlled trial.

This systematic review performed a systematic methodological quality assessment of the 26 included studies [30-55], revealing that most research performed well across key evaluation domains (Figure 2 [30-55] and Figure 3). Evaluation across five dimensions, including the randomization process, deviations from intended interventions, missing outcome data, measurement of the outcome, and selection of the reported results, found that most studies were overall rated as low risk, with single uncertainties appearing only in the domain of deviations from intended interventions. Specifically, studies such as Alley et al (2022) [45], Brickwood et al (2021) [36], Cai et al (2022) [48], Li et al (2025) [33], Oliveira et al (2024) [43], and Yates et al (2009) [54] provided detailed descriptions of random sequence generation and allocation concealment, and used objective tools such as accelerometers or pedometers to avoid measurement bias potentially arising from nonblinded designs. Meanwhile, Delbaere et al (2021) [35], Kawaguchi et al (2024) [52], Lee et al (2025) [38], Recio-Rodríguez et al (2022) [55], Uemura et al (2024) [47], and Mouton et al (2015) [39] further ensured the robustness of the overall evidence quality through comprehensive protocol registration and transparent results reporting, reflecting the rigor of current DHI research designs.

Some studies were rated as having some concerns or high risk due to simultaneous methodological limitations across multiple dimensions. Roberts et al (2019) [44], Muellmann et al (2019) [53], and Bickmore et al (2013) [50] were marked as having some concerns in both randomization and intervention deviation due to unclear allocation concealment details and the inability to blind the intervention process, resulting in a downgrade of the overall rating. Additionally, Ashe et al (2015) [32], Kwan et al (2020) [51], Pischke et al (2022) [40], and Van Dyck et al (2019) [34] accumulated multiple bias risks in data integrity due to high attrition rates or lack of clear intention-to-treat analysis for missing data, and were overall judged as having some concerns. Regarding studies judged as high risk, Zhou et al (2021) [41] showed significant defects in the missing outcome data domain, with a high proportion of participant dropout in the later stages without reasonable explanation, reducing evidence reliability. Audsley et al (2020) [30] demonstrated a high risk of bias in the selection of the reported results, as the final reported indicators deviated significantly from the prespecified trial registration protocol; this tendency for selective reporting directly pushed this systematic review’s overall risk rating to high, suggesting caution when interpreting its pooled effects.

### Meta-Analysis Pooled Results

#### Meta-Analysis of the Effects of Multicomponent DHIs on Daily Steps in Older Adults

The meta-analysis results showed that among the 14 included studies (n=1651), multicomponent DHIs significantly improved daily steps in older adults [32,36,41-44,46,48-52,54,55]. The weighted pooled mean estimate for daily steps merged using the HKSJ random-effects model was 822.8 (95% CI 198.3-1447.3) steps/d, with a statistical test result of *t*_13_=2.85 (*P*=.01). Moderate-to-high statistical heterogeneity existed between studies (*I*^2^=67.5%; *P*<.001). The 95% PI calculated based on the Nagashima method was −1452.4 to 3098.0 steps/d. This PI crossed the line of no effect, quantifying the potential distribution range of the true effect size in future similar clinical settings and suggesting a certain degree of volatility in intervention effects across different research contexts. To evaluate the robustness of the results, this systematic review conducted multidimensional sensitivity analyses (Table S1 in Multimedia Appendix 2). The “leave-one-out” analysis showed that the pooled MD values after excluding studies one by one fluctuated between 677.84 and 938.34 steps/d, and the 95% CI of all iteration results did not cross 0. The analysis identified that Zhou et al (2021) [41] and Yates et al (2009) [54] contributed significantly to the model heterogeneity; excluding Zhou et al (2021) [41] reduced *I*^2^ to 60.1%. Furthermore, sensitivity analysis based on the SMD (Hedges *g*) further confirmed the robustness of the results. The pooled effect size merged using the HKSJ random-effects model was 0.4 (95% CI 0.1 to 0.6; *t*_13_=3.44; *P*=.004), and its heterogeneity level (*I*^2^=68.7%) was highly consistent with the primary analysis results. The calculated 95% PI was −0.4 to 1.1. The MD and SMD results exhibited consistency in effect direction and significance (Figure S1 in Multimedia Appendix 2).

### Meta-Analysis of the Effects of Multicomponent DHIs on MVPA in Older Adults

The meta-analysis results indicated that among the 13 included studies (n=1523), multicomponent DHIs increased MVPA duration in older adults [32-34,37,38,40,41,45-47,49,51,53]. The weighted pooled mean estimate merged using the HKSJ random-effects model was 45.9 (95% CI 23.9-67.9) min/wk, with a statistical test result of *t*_12_=4.1 (*P*<.001). The statistical heterogeneity test between studies showed *I*^2^=18.6% (*P*=.26). The 95% PI calculated based on the Nagashima correction was −9.4 to 101.2 min/wk, quantifying the distribution range of true effect sizes in similar clinical settings. Sensitivity analysis addressing the diversity of measurement tools in the original studies showed that the pooled effect size calculated based on the SMD was 0.3 (95% CI 0.2-0.5; *t*_12_=4.2; *P*<.001), with an interstudy heterogeneity level of *I*^2^=18.2%. The calculated 95% PI was −0.1 to 0.7 (Figure S2 in Multimedia Appendix 2). The results showed that the summary statistics expressed as MD and SMD demonstrated consistency in effect direction and significance levels.

### Meta-Analysis of the Effects of Multicomponent DHIs on Weekly Sedentary Time in Older Adults

The meta-analysis results showed that among the 5 included studies (n=741), the effect of multicomponent DHIs on weekly SB in older adults did not reach statistical significance [33,40,44,45,53]. The weighted pooled mean estimate merged using the HKSJ random-effects model was −283.7 (95% CI −610.8 to 43.5) min/wk, with a statistical test result of *t*_13_=−2.41 (*P*=.07). Significant statistical heterogeneity existed between studies (*I*^2^=76.2%; *P*=.002). The calculated 95% PI was −984.5 to 417.1 min/wk. This indicator quantified the distribution range of true effect sizes in future similar intervention scenarios, suggesting that intervention effects fluctuate considerably across different individuals and research contexts.

### Meta-Analysis of the Effects of Multicomponent DHIs on Weekly TPA Duration in Older Adults

The meta-analysis results showed that among the 7 included studies (n=886) [32,34,39,42,45,47,49], the effect of multicomponent DHIs on weekly TPA duration in older adults did not reach statistical significance [34,39,42,45,47,49,51]. The weighted pooled mean estimate merged using the HKSJ random-effects model was 104.4 (95% CI −109.2 to 318.0) min/wk, with a statistical test result of *t*_6_=1.20 (*P*=.28). Moderate statistical heterogeneity existed between studies (*I*^2^=55.3%; *P*=.04). The calculated 95% PI was −444.4 to 653.2 min/wk. This indicator quantified the distribution range of true effect sizes in similar clinical settings.

### Meta-Analysis of the Effects of Multicomponent DHIs on Weekly LPA Duration in Older Adults

The meta-analysis results indicated that among the 4 included studies (n=1153) [33,35,47,49], the effect of multicomponent DHIs on weekly LPA duration in older adults did not reach statistical significance [33,47,49,56]. The weighted pooled mean estimate merged using the HKSJ random-effects model was 39.3 (95% CI −96.2 to 174.7) min/wk, with a statistical test result of *t*_3_=0.92 (*P*=.42). Moderate-to-high statistical heterogeneity existed between studies (*I*^2^=60.3%; *P*=.06). The calculated 95% PI was −227.6 to 306.2 min/wk. This indicator quantified the distribution range of true effect sizes in similar clinical settings.

### Subgroup Analysis

#### Subgroup Analysis of the Effects of Multicomponent DHIs on Daily Steps in Older Adults

The subgroup analysis results for daily steps are summarized in Table 2. Regarding intervention duration, the pooled MD for short-term interventions (≤12 wk, k=6) and medium-to-long-term interventions (>12 wk, k=8) were 789.6 (95% CI −941.2 to 2520.5; 95% PI −3414.0 to 4993.3) and 838.6 (95% CI 208.6-1468.6; 95% PI −1020.9 to 2698.1), respectively. The test for subgroup differences showed *P*=.95, suggesting that intervention duration is not a key variable moderating step-count outcomes (Figure S3 in Multimedia Appendix 2). Regarding population characteristics, the difference between groups was statistically significant (*P*=.049), with the intervention effect in the high-risk or specific population group (MD=1256.1, 95% CI 258.6-2253.6) being significantly higher than that in the general healthy or community population group (MD=260.5, 95% CI −459.6 to 980.5). This reflects that DHIs may be more effective in motivating populations with specific health needs (Figure S4 in Multimedia Appendix 2). Regarding control group type, the pooled MD for the active control group (k=10) and the routine care group (k=4) were 599.0 (95% CI −101.0 to 1299.1) and 1478.1 (95% CI −674.6 to 3630.9), respectively. Although the routine care group numerically showed a higher increase in steps, the test for subgroup differences showed *P*=.24, and heterogeneity levels were highly similar between the 2 groups (*I*^2^ of 65.6% and 66.1%, respectively), indicating that the control setting has limited moderating effect on step-count intervention outcomes (Figure S5 in Multimedia Appendix 2).

**Table 2.** Summary table evaluating the effects of multicomponent digital health interventions on daily steps (steps/d) in older adults across different subgroup characteristics based on the HKSJ^a^ random-effects model.

Subgroup classification	k	Pooled MD^b^ (95% CI)		95% PI^c^	*I*^2^ (%)	*P*_subgroup_
Duration						.95
Short-term (≤12 wk)	6	789.6 (−941.2 to 2520.5)		−3414.0 to 4993.3	77.3	
Medium-to-long-term (>12 wk)	8	838.6 (208.6 to 1468.6)		−1020.9 to 2698.1	60.8	
Health status						.049^d^
General healthy/community-dwelling	6	260.5 (−459.6 to 980.5)		−1376.5 to 1897.4	44.3	
High-risk/specific populations	8	1256.1 (258.6 to 2253.6)		−1587.4 to 4099.6	76	
Control group setting						.24
Active control/health education	10	599 (−101.0 to 1299.1)		−1731.0 to 2929.1	65.6	
Routine care/basic monitoring	4	1478.1 (−674.6 to 3630.9)		−3819.5 to 6775.8	66.1	
Technical carrier						.2
Mobile app/interactive platform	9	618.4 (−305.4 to 1542.2)		−2340.5 to 3577.3	67.9	
Standalone wearable device	5	1307.3 (299.1 to 2315.4)		−750.8 to 3365.3	31.4	
Delivery agent						.21
Technology-driven	7	459.9 (−381.1 to 1300.9)		−2040.6 to 2960.3	58.4	
Technology-mediated	7	1185.5 (43.4 to 2327.6)		−2150.3 to 4521.3	72.1	

a HKSJ: Hartung-Knapp-Sidik-Jonkman.

b MD: mean difference.

c PI: prediction interval.

d *P* values <.05 were considered statistically significant.

Regarding technical carriers, the pooled MD for the standalone wearable device group (1307.3, 95% CI 299.1-2315.4) was numerically superior to the mobile app or interactive platform group (618.4, 95% CI −305.4 to 1542.2), and its intragroup heterogeneity decreased to 31.4% (subgroup *P*=.20), suggesting this technical carrier is an important factor in explaining daily step-count heterogeneity (Figure S6 in Multimedia Appendix 2). Finally, regarding technical implementation mode, the pooled MD was 459.9 (95% CI −381.1 to 1300.9) for technology-driven interventions (k=7) and 1185.5 (95% CI 43.4 to 2327.6) for technology-mediated interventions (k=7; subgroup *P*=.21). Although the difference did not reach significance, the technology-mediated type demonstrated stronger statistical significance (*P*=.046; Figure S7 in Multimedia Appendix 2).

### Subgroup Analysis of the Effects of Multicomponent DHIs on MVPA in Older Adults

The results of the subgroup analysis for MVPA are summarized in Table 3. Regarding the dimension of intervention duration, the pooled MD values for short-term interventions (≤12 wk, k=7) and medium-to-long-term interventions (>12 wk, k=6) were 53.2 (95% CI 24.2-82.2; 95% PI −1.9 to 108.3) and 41.0 (95% CI 4.6-77.4; 95% PI −40.3 to 122.3), respectively. The test for subgroup differences showed *P*=.55, with intragroup heterogeneity of *I*^2^=0% and *I*^2^=24.8%, respectively (Figure S8 in Multimedia Appendix 2). Regarding population characteristics, the pooled MD for the general healthy or community-dwelling older adult group (k=7) and the high-risk or specific population group (k=6) were 40.1 (95% CI 6.1-74.0; 95% PI −40.6 to 120.7) and 57.0 (95% CI 28.8-85.1; 95% PI 14.9-99.0), respectively. The test for subgroup differences showed *P*=.45, with intragroup heterogeneity of *I*^2^=27.2% and *I*^2^=0%, respectively (Figure S9 in Multimedia Appendix 2). Regarding control group type, the pooled MD for the active control group (k=7) and the routine care group (k=6) were 39.6 (95% CI 3.3-76.0; 95% PI −41.7 to 120.9) and 54.3 (95% CI 28.2-80.4; 95% PI 5.1-103.5), respectively; no statistically significant difference was observed between the two groups (*P*=.52), with intragroup heterogeneity of *I*^2^=14.3% and *I*^2^=0%, respectively (Figure S10 in Multimedia Appendix 2).

**Table 3.** Summary table evaluating the effects of multicomponent digital health interventions on weekly moderate-to-vigorous physical activity time (min/wk) in older adults across different subgroup characteristics based on the HKSJ^a^ random-effects model.

Subgroup classification	k	Pooled MD^b^ 95% CI		95% PI^c^	*I*^2^ (%)	*P*_subgroup_
Duration						.55
Short-term (≤12 wk)	7	53.2 (24.2 to 82.2)		−1.9 to 108.3	0	
Medium-to-long-term (>12 wk)	6	41 (4.6 to 77.4)		−40.3 to 122.3	24.8	
Health status						.45
General healthy/community-dwelling	7	40.1 (6.1 to 74.0)		−40.6 to 120.7	27.2	
High-risk/specific populations	6	57 (28.8 to 85.1)		14.9 to 99.0	0	
Control group setting						.52
Active control/health education	7	39.6 (3.3 to 76.0)		−41.7 to 120.9	14.3	
Routine care/basic monitoring	6	54.3 (28.2 to 80.4)		5.1 to 103.5	0	
Technical carrier						.66
Mobile app/interactive platform	10	52 (28.1 to 76.0)		8.1 to 96.0	0	
Standalone wearable device	3	38.8 (−78.0 to 155.6)		−234.3 to 311.9	38.6	
Delivery agent						.25
Technology-driven	6	58.3 (16.6 to 100.1)		−23.4 to 140.0	3.2	
Technology-mediated	7	36.8 (13.1 to 60.4)		−23.4 to 97.0	20.9	

a HKSJ: Hartung-Knapp-Sidik-Jonkman.

b MD: mean difference.

c PI: prediction interval.

Regarding the dimension of technical carriers, the pooled MD for the mobile app or interactive platform group (k=10) was 52.0 (95% CI 28.1-76.0; 95% PI 8.1-96.0), while the pooled MD for the standalone wearable device group (k=3) was 38.8 (95% CI −78.0-155.6; 95% PI −234.3 to 311.9). The test for subgroup differences showed *P*=.66, with *I*^2^ values of 0% and 38.6%, respectively (Figure S11 in Multimedia Appendix 2). Finally, regarding the dimension of technical implementation mode, the pooled MD for the technology-driven type (k=6) was 58.3 (95% CI 16.6-100.1; 95% PI −23.4 to 140.0), and the pooled MD for the technology-mediated type (k=7) was 36.8 (95% CI 13.1-60.4; 95% PI −23.4 to 97.0). The test for subgroup differences showed *P*=.25, with intragroup heterogeneity of *I*^2^=3.2% and *I*^2^=20.9%, respectively (Figure S12 in Multimedia Appendix 2).

### Meta-Regression Analysis

**Figure 4.**
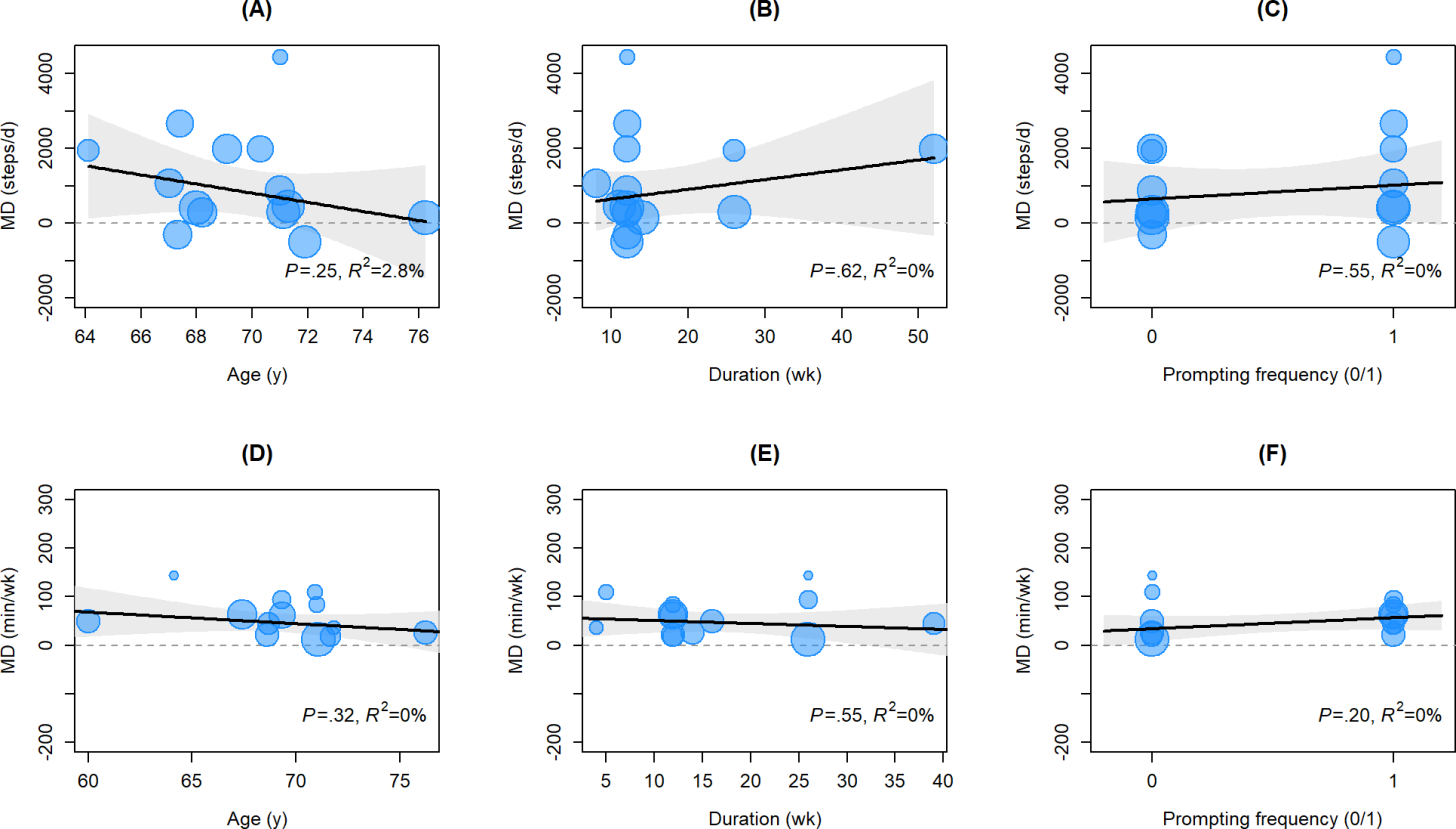
Univariate meta-regression scatter plots evaluating the effects of participants’ mean age, total intervention duration, and intervention prompt frequency on daily steps and weekly MVPA in multicomponent digital health interventions. Each bubble in the figure represents an original study, with the bubble size proportional to the weight assigned to that study in the regression analysis (typically the inverse of the sample size). The solid center line represents the fitted meta-regression line, reflecting the correlation trend between the independent variable and the effect size. The shaded area represents the 95% CI of the regression line. *P* values are used to determine whether the regression coefficients are statistically significant. MD: mean difference; MVPA: moderate-to-vigorous physical activity.

For the daily steps indicator, the meta-regression analysis included 14 studies (k=14) [32,36,40-46,48-51,54,55]. The results indicated that none of the 3 examined covariates could significantly explain the interstudy heterogeneity (Figure 4). Specifically, participants’ mean age (Figure 4A: *P*=.25; *R*^2^=2.84%; τ^2^=8.25 times 105), intervention duration (Figure 4B: *P*=.615; *R*^2^=0%; τ^2^=1.07 times 106), and prompt frequency (Figure 4C: *P*=.55; *R*^2^=0%; τ^2^=1.08 times 106) showed no significant linear correlation with the postintervention increment in steps. The regression lines appeared flat, with observed values from individual studies distributed on both sides of the lines. Influence diagnostics for these models (detailed in Figures S13-S15 in Multimedia Appendix 2) showed that Cook distance for all included studies remained at low levels, and no strong influential points significantly driving the regression trajectories were found. This suggests that the effect of multicomponent DHIs on increasing daily steps maintained good stability across different age groups, implementation periods, and reminder intensities.

For the MVPA indicator, the meta-regression analysis covered 13 studies (k=13) [32,34,37,38,40,41,43,45-47,49,51,53]. The results demonstrated that the intervention effect for this indicator was likewise not significantly influenced by the preset moderators. Participants’ age (Figure 4D: *P*=.32; *R*^2^=0%; τ^2^=601.43), intervention duration (Figure 4E: *P*=.55; *R*^2^=0%; τ^2^=614.94), and prompt frequency (Figure 4F: *P*=.20; *R*^2^=0%; τ^2^=574.23) all failed to reach statistical significance. Influence diagnostics identified a few studies with relatively high Cook distances (detailed in Figures S16-S18 in Multimedia Appendix 2), but after applying the Sidik-Jonkman estimator and Hartung-Knapp adjustment, the driving effect of these strong influential points on the regression slopes was effectively suppressed. Although some residual heterogeneity (τ^2^) remained in the models, the *R*^2^ values were all 0%, indicating that the variation in MVPA effect sizes could not be explained by the aforementioned variables.

### Analysis of Small-Study Effects and Robustness Testing

**Figure 5.**
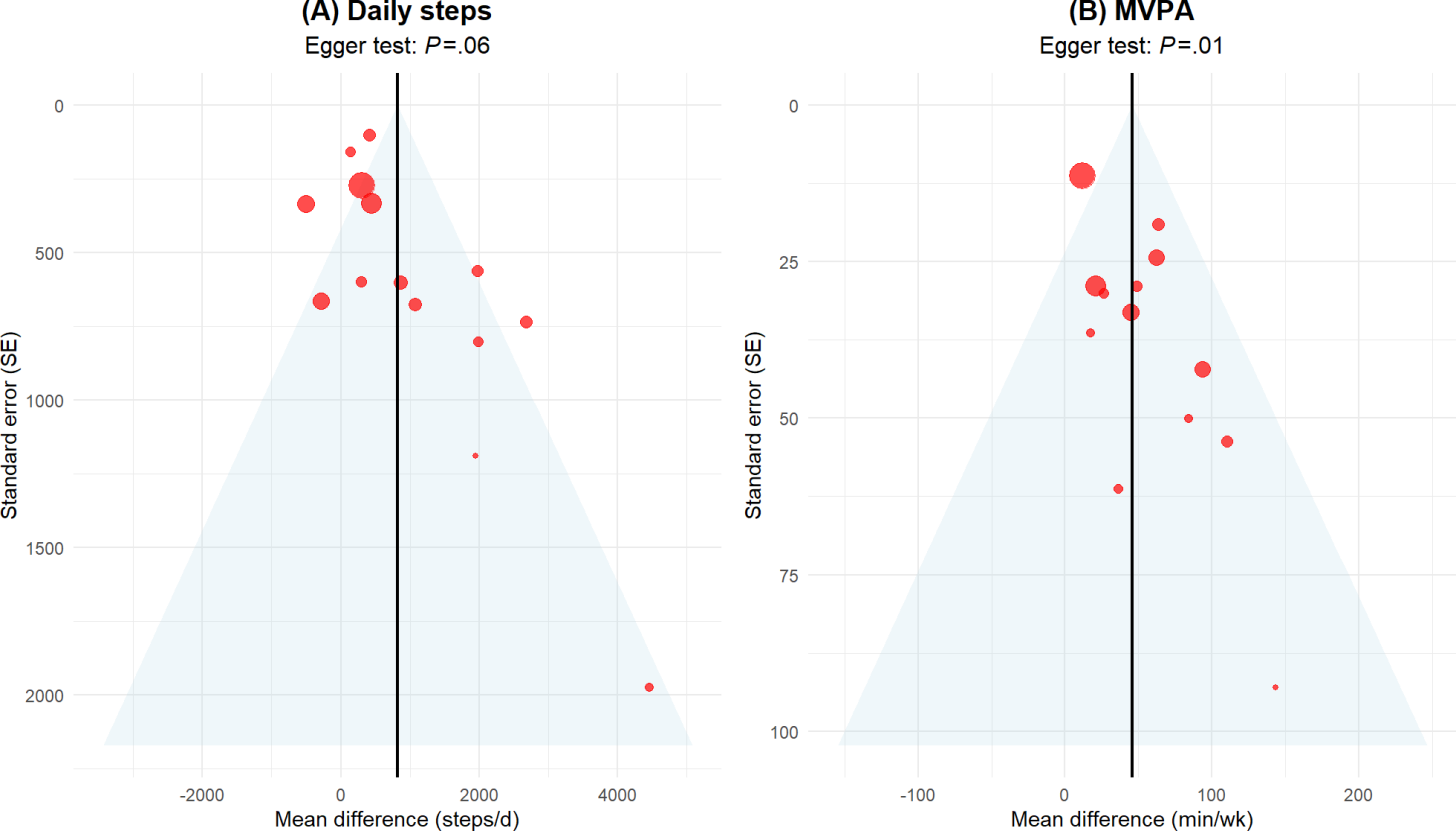
Egger linear regression test funnel plots of the effects of multicomponent digital health interventions on daily steps and weekly MVPA in older adults. Each scatter point in the figure represents an original study. Given that this systematic review included a large total sample size, the distribution of the funnel plot can more reliably reflect the authenticity of the evidence. The horizontal axis represents the effect size (mean difference), and the vertical axis represents the SE. The center vertical solid line is the pooled effect size, and the diagonal lines on both sides represent the 95% CIs. The highly symmetrical distribution of points within the funnel, combined with the Egger test *P* value, demonstrates whether significant small-sample effects or publication bias exist. MVPA: moderate-to-vigorous physical activity.

The analysis of small-study effects (Figure 5) showed that for the daily steps indicator, the Egger test result was *P*=.06 (>.05), and the distribution of study points within the funnel plot was basically symmetrical. This suggests that there is no obvious asymmetry caused by small-study effects for this indicator. Conversely, for the MVPA indicator, the Egger test result was *P*=.01 (<.05), and the funnel plot exhibited significant asymmetry. Furthermore, there was a lack of small-sample, negative-result studies distributed to the left of the centerline at the bottom of the funnel. This indicates a significant risk of small-study effects for this indicator, which may stem from publication bias, differences in study quality, or high heterogeneity.

**Figure 6.**
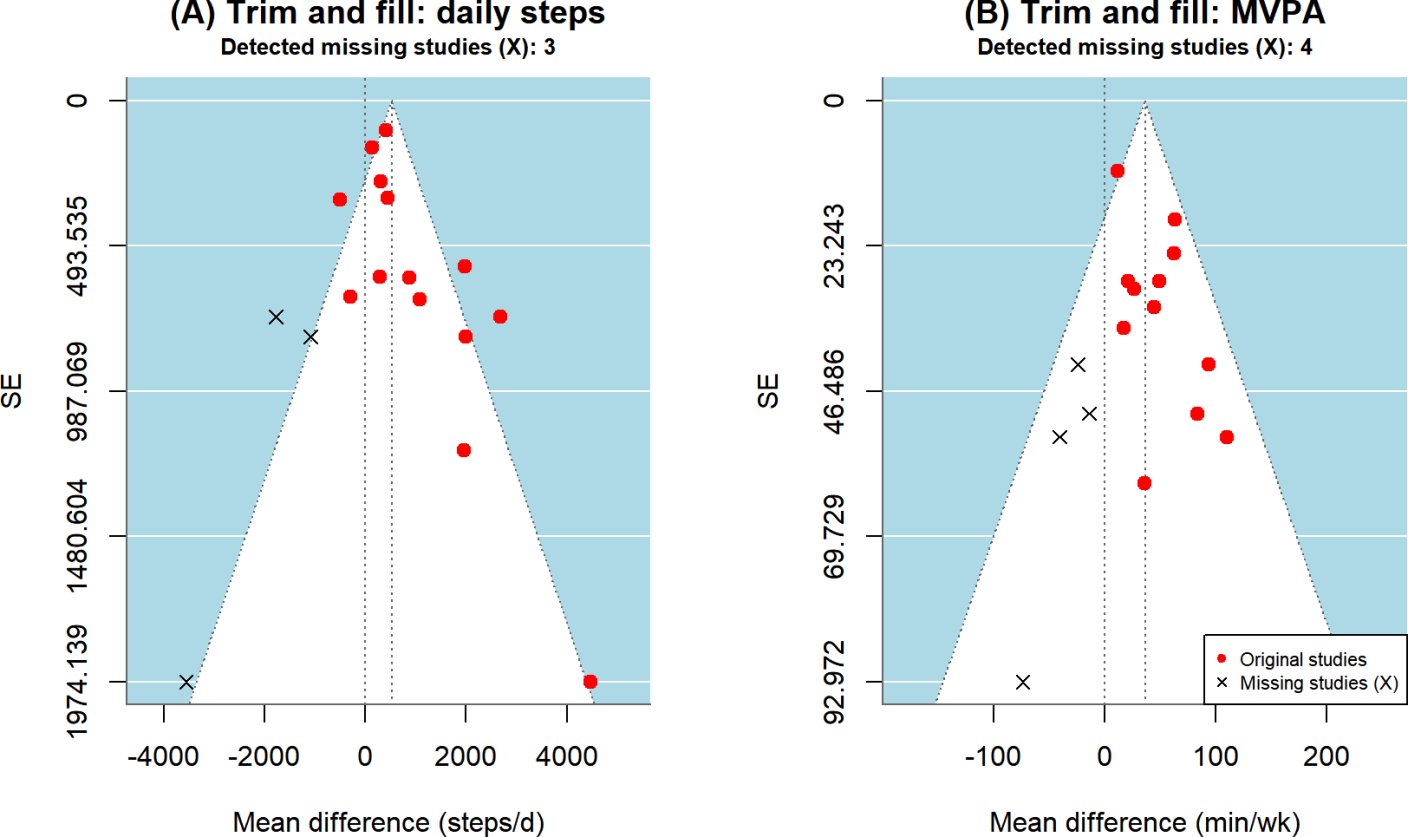
Funnel plots based on the trim-and-fill method correcting for publication bias regarding the effects of digital health interventions on daily steps and weekly MVPA in older adults. MVPA: moderate-to-vigorous physical activity.

Regarding the detected asymmetry, this systematic review used the trim-and-fill method for robustness analysis (Figure 6). For the daily steps indicator, the model identified and filled in 3 potentially missing studies. The adjusted pooled effect size was MD=521.4 (95% CI −239.3 to 1282.0; *P*=.18) steps/d. The results indicate that after considering potential small-study effects, the significant increase in daily steps observed in the original analysis shifted to nonsignificant. For the MVPA indicator, the model identified and filled in 4 potentially missing studies. The adjusted pooled effect size was MD=36.5 (95% CI 11.2-61.8; *P*=.005) min/wk. Although the adjusted effect size decreased compared to the original value (MD=45.9), its statistical significance remained robust. In summary, the distribution after filling was more symmetrical than the original funnel plot. Despite the identified potential asymmetry, MVPA maintained significant clinical meaning after adjustment.

### Evidence Certainty Assessment

This systematic review used the GRADE system to assess the quality of evidence for various indicators of DHIs affecting PA in older adults (Table 4). The assessment results showed that only the quality of evidence for MVPA duration was rated as “moderate.” Although this indicator was downgraded by one level due to significant publication bias (Egger test *P*=.01), its pooled effect remained robust after correction via the trim-and-fill method. In contrast, the quality of evidence for daily steps was rated as “low.” This indicator was downgraded by one level due to significant inconsistency between studies (*I*^2^=67.5%) and further downgraded in the publication bias dimension, because it lost statistical significance during the trim-and-fill stress test (adjusted *P*=.18), suggesting that the results were severely interfered with by small-study effects. Furthermore, the quality of evidence for sedentary time, TPA, and LPA were all rated as “low.” Specifically, sedentary time was downgraded by one level each in the dimensions of inconsistency and imprecision due to extremely high heterogeneity (*I*^2^=76.2%) and a limited number of included studies (k=5). TPA and LPA not only exhibited moderate-to-high heterogeneity (*I*^2^ of 55.3% and 60.3%, respectively), but their pooled effect 95% CIs both crossed the zero line, indicating nonsignificant statistical meaning and a serious risk of imprecision.

**Table 4.** Summary of the quality of evidence grading for outcome indicators (daily steps, MVPA^a^, LPA^b^, TPA^c^, and SB^d^) related to physical activity affected by multicomponent digital health interventions based on the GRADE^e^ framework.

Outcome	Studies (n, k)	Sample size (N)	Effect size (95% CI)	Risk of bias	Inconsistency	Indirectness	Imprecision	Publication bias	Certainty
MVPA time (min/wk)	13	1523	MD^f^ 45.9 (23.9 to 67.9)	Not serious	Not serious	Not serious	Not serious	Serious^g^	Moderate ⊕⊕⊕◯
Daily steps (steps/d)	14	1651	MD 822.8 (198.3 to 1447.3)	Not serious	Serious^h^	Not serious	Not serious	Serious^i^	Low ⊕⊕◯◯
Sedentary time (min/wk)	5	741	MD −283.7 (−610.8 to 43.5)	Not serious	Serious^j^	Not serious	Serious^k^	Not detected	Low ⊕⊕◯◯
TPA (min/wk)	7	886	MD 104.4 (−109.2 to 318.0)	Not serious	Serious^l^	Not serious	Serious^m^	Not detected	Low ⊕⊕◯◯
LPA (min/wk)	4	1153	MD 39.3 (−96.2 to 174.7)	Not serious	Serious^n^	Not serious	Serious^o^	Not detected	Low ⊕⊕◯◯

a MVPA: moderate-to-vigorous physical activity.

b LPA: light physical activity.

c TPA: total physical activity.

d SB: sedentary behavior.

e GRADE: Grading of Recommendations, Assessment, Development, and Evaluation.

f MD: mean difference.

g Publication bias: Egger test showed significant publication bias (*P*=.014); although results remained robust after trim-and-fill correction, the quality was downgraded by one level according to standard protocols.

h Inconsistency: significant heterogeneity existed among studies (*I*2=67.5%), and the prediction interval (95% PI –1452.4 to 3098.0) crossed the null line, indicating substantial variation in intervention effects across studies, thus the quality was downgraded by one level.

i Publication bias: the trim-and-fill stress test showed that the corrected results lost statistical significance (*P*=.18), suggesting the findings were severely affected by small-study effects, therefore the quality was downgraded by one level in the publication bias domain .

j Inconsistency: the original pooled results showed high heterogeneity (*I*2=76.2%) and the prediction interval crossed the zero line, thus the quality was downgraded by one level.

k Imprecision: the number of included studies was small (k=5), the sample size was insufficient, and the 95% CI crossed the null line, indicating a risk of imprecision, therefore the quality was downgraded by one level.

l Inconsistency: moderate-to-high heterogeneity existed (*I*2=55.3%), thus the quality was downgraded by one level.

m Imprecision: the effect size was nonsignificant, its 95% CI crossed the zero line, and the sample size was small, thus the quality was downgraded by one level.

n Inconsistency: moderate heterogeneity existed (*I*2=60.3%), thus the quality was downgraded by one level.

o Imprecision: the effect size was not statistically significant, the CI crossed the zero line, and the sample size was limited, thus the quality was downgraded by one level.

## Discussion

### Main Findings and Evidence Overview

This systematic review evaluated the effects of multicomponent DHIs on PA in older adults through a systematic review and meta-analysis of 26 RCTs. The results found that multicomponent DHI can significantly increase the weekly duration of MVPA in older adults; this result remained robust after correction using the trim-and-fill method and is supported by moderate-quality evidence. In contrast, although the original analysis showed a significant increase in daily steps, the quality of evidence for this indicator was rated as “low” because it lost statistical significance under the trim-and-fill stress test and was affected by significant interstudy heterogeneity. However, due to high heterogeneity and low-quality evidence ratings, it is currently not possible to confirm the statistical significance of DHI in reducing SB, or increasing LPA and TPA. In the subgroup analysis of daily steps, using standalone wearable devices as a technical means showed a superior effect compared to mobile apps or web platforms and significantly reduced intragroup heterogeneity; simultaneously, the effect of interventions on increasing steps in high-risk or specific diseased populations was superior to that in healthy older populations. Other subgroup results for daily steps showed that technology-mediated interventions (involving human participation) and studies using routine care as a control observed larger effect sizes, while different intervention durations (≤12 wk vs >12 wk) showed no significant difference in their impact on step increases. In the subgroup analysis of MVPA, no significant differences in effects were observed across different durations, population characteristics, or technical means, demonstrating good consistency. Meta-regression analysis further confirmed that age, intervention duration, and prompt frequency did not exert significant moderating effects on the effect sizes of steps and MVPA, indicating that after excluding strong influential points, the intervention effect maintained stability across different participant characteristics and implementation periods. Finally, this systematic review clearly distinguished between the average effect and the distribution of effects; although the CIs of core indicators were significant, the PIs for all outcome indicators crossed the line of no effect, reflecting obvious dispersion of intervention effects across different research settings and contexts. This suggests that in future-specific implementation environments, there remains a possibility that interventions may not produce the expected benefits. Therefore, the potential impact of participant characteristics and intervention environments should be fully considered when promoting multicomponent DHI.

### Analysis of Influence Patterns and Moderating Effects of Multicomponent DHIs on Exercise Behavior

The effects of multicomponent DHIs in increasing daily steps and MVPA in older adults stem from the enhancement of participants’ self-efficacy by behavior change techniques, such as real-time monitoring, goal setting, and automated feedback [56]. From a public health perspective, the increase in steps observed in this systematic review (+822.8 steps/d) holds clear clinical benefit value [31]. Previous research has indicated that for the older adult population, an increase of even approximately 500 to 1000 daily steps is significantly associated with a reduced risk of all-cause mortality and can effectively delay the onset of mobility impairment [57]. Meanwhile, the weekly increment of approximately 46 minutes of MVPA reaches nearly one-third of the level recommended by the World Health Organization, which carries important translational medicine significance for improving cardiorespiratory fitness and insulin sensitivity, and reducing cardiovascular disease risk in older adults [58,59]. The step enhancement effect in this systematic review is consistent with the research direction of Wu et al (2023) [18] regarding the use of wearable devices by older adults (SMD 0.59) [18]. In contrast, Wang et al (2025) [15] pointed out that the improvement in steps by application-based interventions (SMD 0.18) was relatively modest, while the promotion effect on MVPA (SMD 0.48) was more prominent [15]. However, when interpreting the findings, a clear distinction must be made between CIs and PIs. Although the CIs for steps and MVPA were significant, their PIs both crossed the null line. This indicates that, influenced by moderate statistical heterogeneity between studies, the intervention effects exhibit significant dispersion across different clinical settings.

This systematic review found that although multicomponent DHIs increased steps and MVPA, their effectiveness in improving SB was limited, which may reflect limitations in the design logic of current intervention protocols [32,39]. Most multicomponent DHI protocols feature monitoring and feedback mechanisms that focus on capturing and motivating “planned exercise” that reaches intensity thresholds, while lacking immediate disruption strategies for prolonged SB [60]. This results in participants being more inclined to increase conscious exercise increments, yet a significant evidence gap remains regarding the reconstruction of all-day sedentary patterns [18]. Furthermore, the lack of a simultaneous significant increase in TPA further reveals the complexity of exercise behavior change in older adults [43]. Despite the significant increase in MVPA, the improvement in overall activity levels remains limited because multicomponent DHIs often lack immediate incentives for trivial, low-intensity daily activities [61,62]. Future protocol designs should optimize reminder strategies [63] and attempt to expand the intervention focus from pure exercise increments to all-day sedentary interruptions, aiming to achieve a more comprehensive reshaping of behavioral patterns in older adults [64]. Additionally, it is noteworthy that the regression coefficients of all meta-regression models were not statistically significant. This result suggests that the preset clinical and methodological covariates do not explain the significant heterogeneity observed between studies; fluctuations in effect size may stem more from deep-level variables that were not captured, such as participants’ baseline digital literacy, the specific interaction logic of the intervention protocol, or the degree of social support [42,65]. Meanwhile, considering the limited number of studies included in the models, insufficient statistical power may have restricted the ability of the models to detect weak moderating effects [66]; therefore, the findings of the meta-regression should be cautiously interpreted as a failure to identify significant moderators within the existing evidence scale. After excluding influential points using the HKSJ model, this finding proves that the positive effects of multicomponent DHIs maintained good stability in the older population aged 60 to 77 years. This challenges the traditional assumption that older age groups inevitably face a “digital divide bottleneck” leading to intervention failure [67], suggesting that as long as the protocol design is reasonable, the response of older adults to core behavior change techniques has cross-population reference value. Subgroup analysis results provide preliminary clues for understanding the heterogeneity of intervention effects. Regarding the daily steps dimension, the technical carrier was identified as a potential source of heterogeneity, with intragroup heterogeneity reduced in the standalone wearable device group. Although the statistical power for testing between-group differences was limited due to the small number of studies within subgroups, standalone wearable devices showed a better effect size trend than the mobile app group, which may be attributed to a more direct interaction method that reduces participants’ dependence on digital literacy [68]. Furthermore, the significant effect on step indicators for populations at high risk of chronic diseases also suggests that when intervention goals are highly correlated with an individual’s sense of health security, multicomponent digital health technology can generate stronger behavioral drivers [69]. Notably, this systematic review also observed opposing trends in the effects of delivery agents across different indicators: step increases leaned toward technology-mediated human support, while MVPA enhancement was more deeply influenced by technology-driven real-time feedback [48,56], further demonstrating the necessity for precision design in multicomponent digital protocols.

Significantly, publication bias tests further revealed the essential difference in evidence certainty between daily steps and MVPA. After applying the trim-and-fill method to adjust for potential small-study effects, the pooled effect for daily steps exhibited clear vulnerability as it lost statistical significance [70]. This finding raises doubts about the robustness of the original effect size for this indicator and directly led to its GRADE evidence quality being downgraded from “moderate” to “low” [24]. In contrast, the intervention effect on MVPA remained robust under stress testing, supporting its evidence-based value as “moderate” quality evidence. This contrast clarifies that the driving effect of multicomponent DHIs on high-intensity exercise behavior in older adults is more certain than simply increasing steps. Combined with the GRADE evidence quality assessment, although the presence of heterogeneity lowered the certainty rating of some outcome indicators, the observed effect sizes reached the aforementioned clinical benefit benchmarks, making their reference value for clinical decision-making remain clear [31,57]. When discussing the sustainability of intervention effects, it must be clarified that the current meta-regression reflects the stability of effect sizes during the intervention implementation period. However, in the current digital health field, distinguishing between “effectiveness during long-term intervention” and “effect maintenance after intervention withdrawal” is crucial, and the latter still faces a significant research evidence gap in existing literature [47,48]. This inference suggests that future research should focus on how to transform digital external incentives into participants’ lasting internal behavioral inertia [71] and conduct age-friendly modifications of digital interfaces for older adults to maintain participation momentum, which still requires more long-term tracking studies for confirmation [72].

### Practical Implications

This systematic review provides evidence-based support for the management of community-dwelling older adults’ health and the development of multicomponent digital health exercise intervention protocols. At the public health policy level, multicomponent DHIs can serve as an effective supplement to traditional face-to-face interventions; particularly in resource-limited community settings, their features of wide coverage and remote support offer high cost-effectiveness [36]. Given the significant benefits observed among populations at high risk of chronic diseases, it is recommended that multicomponent digital health monitoring measures be positioned forward into the risk screening stage, aiming to delay the transition of older adults toward pathological states through early behavioral interventions [33]. The practical path should not only pursue an increase in exercise volume but should also attempt to guide older adults to reduce SB by increasing light activities such as walking and housework. This approach is more consistent with the physical condition of the advanced older adults and is easier to maintain over the long term than simply pursuing high-intensity exercise goals [73]. However, given that the PIs for the core indicators in this systematic review all crossed the null line, relevant institutions must remain cautious when translating the evidence-based findings of this systematic review. The effectiveness of multicomponent DHIs highly depends on the specific implementation environment, the intensity of technical support, and the digital literacy of the participants; in contexts lacking resources or necessary human assistance, there is a risk that intervention effects may fail to reach targets [74]. Therefore, practice should emphasize personalized adaptation based on local conditions.

In terms of technical carrier selection, the efficacy advantages demonstrated by standalone wearable devices suggest that the research and development of age-friendly digital products should prioritize simplifying human-computer interaction logic. Compared to mobile apps that require higher digital skills, hardware carriers with passive monitoring processes and intuitive feedback are more aligned with the operating habits of the older population [36]. Addressing the phenomenon of diminishing effects in advanced older individuals, product design needs to provide differentiated interface displays and guidance modes [10]. Simultaneously, intervention protocols should focus on the trend of effect attenuation over extended cycles [75]. Clinicians and managers need to dynamically adjust goal settings based on participant feedback, and be timely in introducing social support or professional involvement to mitigate the decline in adherence during long-term application [42]. Furthermore, intervention protocols should break through single step-count assessments and emphasize diversified goals such as “sedentary interruptions” and the “embedding of trivial physical activities,” achieving a comprehensive reshaping of the daily behavior patterns of older adults [46].

### Research Limitations and Future Outlook

Although this systematic review evaluated the efficacy of multicomponent DHIs through multidimensional indicators, some limitations remain in terms of methodology and evidence application. Since most included studies combined digital tools with human support, the independent contribution of digital technology remains uncertain [31,36]. At the study design level, the specificity of digital interventions meant that most original studies could not achieve double-blinding of participants and implementers, increasing the risk of performance bias [39,46]. Furthermore, the attribution analysis of intervention effects is similarly limited; because most protocols adopted a multicomponent model, with differences in core interaction agents compared to auxiliary monitoring tools and the intensity of accompanying human support. The complexity of these components makes it difficult to completely isolate the respective contributions of the automated features of the digital technology itself from the accompanying social support or professional guidance [39,76]. Meanwhile, the significant variation in control group settings, ranging from passive waiting to active health education, may have diluted the estimation of the “incremental value” of digital technology [32,47]. From a systemic perspective of behavior monitoring, this systematic review was not yet able to fully incorporate the daily behaviors of older adults into a 24-hour PA comprehensive framework; since the total daily time for older adults is constant, changes in specific exercise indicators caused by digital interventions are inevitably accompanied by the reallocation of sleep or trivial daily activities [77,78], and this systematic review struggled to reveal the deep impact of digital interventions on all-day time-use patterns. Additionally, considering that the follow-up periods of most studies coincided with the intervention cycles, the effects revealed by this systematic review primarily reflect immediate efficacy during the implementation period; as the original literature generally lacks long-term tracking data after the cessation of interventions, it remains difficult to confirm whether digital interventions can be transformed into the maintenance of lasting behavioral habits once technical support is removed [33,41]. Although this systematic review covered multiple indicators, the number of studies targeting LPA and TPA was relatively small, which perhaps resulted in insufficient statistical power for the pooled effect sizes. Furthermore, differences existed among original studies in the choice of PA measurement tools, including both objective monitoring devices and self-report questionnaires; this inconsistency in measurement methods may have introduced a degree of heterogeneity during the data pooling process [35,44]. The participant populations were mainly concentrated on healthy community-dwelling older adults or groups at high risk of chronic diseases; therefore, caution should be exercised when extrapolating the conclusions to groups with severe functional impairments or specific clinical conditions [36,46]. Although the results showed that the moderating effects of age, intervention duration, and prompting frequency were all nonsignificant, this may be limited by insufficient statistical power due to the small number of included studies. Moreover, the residual heterogeneity (τ^2^) still present in the models suggests that unobserved variables, such as baseline digital skills and socioeconomic status, may still contribute potential influences [79].

Based on the aforementioned limitations, future research needs to deepen in the following directions. First, emphasis should be placed on distinguishing “effectiveness during the intervention period” from “effect maintenance after intervention withdrawal,” evaluating the potential of digital technology in promoting the long-term sustainability of behavior change in older adults through long-term follow-up after a washout period [41]. Given the trend of effect attenuation that may occur as cycles extend, exploring dynamic feedback mechanisms with lasting driving force and age-friendly interface optimization for the advanced older adults is crucial [75]. Second, subsequent studies should actively adopt 24-hour PA comprehensive guidelines to explore how multicomponent digital health technology can optimize health outcomes for older adults through more scientific time reallocation strategies by simultaneously monitoring sleep, SB, and PA of various intensities [78]. Regarding the complexity of intervention components, it is recommended to conduct head-to-head studies or component-dismantling trials to clarify the efficacy differences between automated technology components and different intensities of human support [80]. Additionally, conducting precise exercise prescription research targeting specific health risk subgroups will help provide more systemic evidence-based support for the digital health management of older adults [81].

### Conclusions

This review systematically evaluated the impact of multicomponent DHIs on the PA behavior of older adults. The research suggests that digital interventions may effectively improve daily steps and MVPA levels in older adults, but there is currently no significant statistical evidence to support their role in reducing sedentary time or improving LPA. Since most original studies combined digital tools with human support, the independent driving role of digital technology within these interventions requires further verification. Meta-regression and subgroup analyses further revealed that intervention effects maintained good stability across different age gradients, intervention cycles, and frequencies, and that standalone wearable devices possessed an efficacy advantage over mobile apps in driving behavior change in older adults. Unlike previous reviews focusing on general adult populations or single exercise indicators, this systematic review focuses on the uniqueness of the older population; through in-depth comparison with existing evidence, this systematic review not only verifies the immediate effectiveness of multicomponent digital interventions, but also evaluates the distribution of effects in real-world settings through more cautious statistical models, identifying that intervention benefits possess clear uncertainty across different contexts. This systematic review brings key evidence regarding the good adaptability of the older population to multicomponent digital interventions, suggesting that advanced age does not significantly weaken the efficacy of technical interventions, and clearly identifies “effect maintenance after intervention withdrawal” as an urgent gap to be filled in the current evidence chain. In terms of real-world significance, this systematic review confirms that promoting hardware carriers with simple interaction and high automation is an effective path for reshaping health behavior patterns in older adults, providing core evidence-based guidance for formulating low-cost, broad-coverage, and high-adherence precise public health intervention strategies in an aging society.

## Supplementary material

10.2196/91338 Multimedia Appendix 1 Comprehensive search strategies and Boolean logic for all 5 databases updated as of February 20, 2026, including the detailed deduplication process.

10.2196/91338 Multimedia Appendix 2 Evidence and robustness analyses, including leave-one-out sensitivity analysis, HKSJ random-effects forest plots with 95% PIs for primary and subgroup outcomes, and meta-regression influence diagnostics. HKSJ: Hartung-Knapp-Sidik-Jonkman; PI: prediction interval.

10.2196/91338 Checklist 1 PRISMA 2020 checklist which specifies the precise manuscript page and line numbers where each of the 27 reporting items—from title and abstract to methods, results, and discussion—is addressed.

10.2196/91338 Checklist 2 PRISMA 2020 for Abstracts checklist, which confirms that the study's abstract follows the 12-item reporting standard covering essential elements such as eligibility criteria, synthesis of results, and funding information.

10.2196/91338 Checklist 3 PRISMA-S checklist which details the reporting of literature search strategies across 16 items, specifically mapping information sources, search strings, and deduplication processes to their respective locations in the manuscript and Multimedia Appendix 1.

## Abbreviations

DHI: digital health intervention
GRADE: Grading of Recommendations, Assessment, Development, and Evaluation
HKSJ: Hartung-Knapp-Sidik-Jonkman
LPA: light physical activity
MD: mean difference
MeSH: Medical Subject Headings
MVPA: moderate-to-vigorous physical activity
PA: physical activity
PI: prediction interval
PICOS: Participants, Interventions, Comparisons, Outcomes, Study Type
PRISMA: Preferred Reporting Items for Systematic Reviews and Meta-Analyses
PRISMA-S: Preferred Reporting Items for Systematic Reviews and Meta-Analyses literature search extension
RCT: randomized controlled trial
SB: sedentary behavior
SMD: standardized mean difference
TIAB: title and abstract
TPA: total physical activity
TS: topic
XB: exact bound
