# Supplementary material for: Effects of Multicomponent Digital Health Interventions on Multidimensional Physical Activity in Older Adults: Systematic Review, Meta-Analysis, and Meta-Regression of Randomized Controlled Trials
Source: J Med Internet Res. 2026 May 29;28:e91338. doi: 10.2196/91338 (PMC13221160; doi:10.2196/91338)
Supplement: Multimedia Appendix 1 — Comprehensive search strategies and Boolean logic for all 5 databases updated as of February 20, 2026, including the detailed deduplication process. [file jmir-v28-e91338-s001.docx]

**Search Strategies**

**Database: PubMed (via NCBI)**

**Date of Search: February 20, 2026**

| **Step** | **Search Terms** | **Results** |
| --- | --- | --- |
| #1 | "Aged"[Mesh] OR "Aged, 80 and over"[Mesh] | 3,833,888 |
| #2 | (older adult*[Tiab] OR elderly[Tiab] OR senior*[Tiab] OR geriatric*[Tiab] OR aging[Tiab] OR ageing[Tiab]) | 874,645 |
| #3 | #1 OR #2 | 4,265,190 |
| #4 | "Telemedicine"[Mesh] OR "Mobile Applications"[Mesh] OR "Smartphone"[Mesh] OR "Wearable Electronic Devices"[Mesh] OR "Fitness Trackers"[Mesh] | 101,785 |
| #5 | (digital health[Tiab] OR mHealth[Tiab] OR eHealth[Tiab] OR telehealth[Tiab] OR mobile app*[Tiab] OR wearable*[Tiab] OR smartphone*[Tiab] OR pedometer*[Tiab] OR activity tracker*[Tiab] OR internet-based[Tiab] OR web-based[Tiab] OR online intervention*[Tiab] OR text messag*[Tiab]) | 206,055 |
| #6 | #4 OR #5 | 253,900 |
| #7 | "Exercise"[Mesh] OR "Motor Activity"[Mesh] OR "Sedentary Behavior"[Mesh] OR "Walking"[Mesh] | 414,382 |
| #8 | (physical activity[Tiab] OR exercise*[Tiab] OR walking[Tiab] OR daily steps[Tiab] OR step count*[Tiab] OR MVPA[Tiab] OR sedentary[Tiab] OR sitting time[Tiab] OR acceleromet*[Tiab] OR active lifestyle[Tiab]) | 664,371 |
| #9 | #7 OR #8 | 837,591 |
| #10 | "Randomized Controlled Trial"[Publication Type] | 1,360,836 |
| #11 | (randomized[Tiab] OR randomised[Tiab] OR RCT[Tiab] OR random allocation[Tiab] OR clinical trial*[Tiab]) | 655,739 |
| #12 | #10 OR #11 | 1,570,488 |
| #13 | #3 AND #6 AND #9 AND #12 | 2,071 |

**Database: Web of Science Core Collection**

**Date of Search: February 20, 2026**

| **Step** | **Search Terms** | **Results** |
| --- | --- | --- |
| #1 | TS=("older adult*" OR elderly OR senior* OR geriatric* OR aging OR ageing) | 4,064,560 |
| #2 | TS=("digital health" OR mHealth OR eHealth OR telehealth OR "mobile app*" OR wearable* OR smartphone* OR pedometer* OR "activity tracker*" OR "internet-based" OR "web-based" OR "online intervention*" OR "text messag*") | 378,574 |
| #3 | TS=("physical activity" OR exercise* OR walking OR "daily steps" OR "step count*" OR MVPA OR sedentary OR "sitting time" OR acceleromet* OR "active lifestyle") | 916,098 |
| #4 | TS=("randomized controlled trial" OR randomized OR randomised OR RCT OR "random allocation" OR "clinical trial*") | 1,446,851 |
| #5 | #1 AND #2 AND #3 AND #4 | 2,710 |

**Database: Embase (via Elsevier)**

**Date of Search: February 20, 2026**

| **Step** | **Search Terms** | **Results** |
| --- | --- | --- |
| #1 | aged'/exp | 4,659,922 |
| #2 | older adult*':ti,ab OR elderly:ti,ab OR senior*:ti,ab OR geriatric*:ti,ab OR aging:ti,ab OR ageing:ti,ab | 1,144,819 |
| #3 | #1 OR #2 | 5,163,254 |
| #4 | telemedicine'/exp OR 'mobile application'/exp OR 'smartphone'/exp OR 'wearable computer'/exp OR 'fitness tracker'/exp | 177,852 |
| #5 | digital health':ti,ab OR mhealth:ti,ab OR ehealth:ti,ab OR telehealth:ti,ab OR 'mobile app*':ti,ab OR wearable*:ti,ab OR smartphone*:ti,ab OR pedometer*:ti,ab OR 'activity tracker*':ti,ab OR 'internet-based':ti,ab OR 'web-based':ti,ab OR 'online intervention*':ti,ab OR 'text messag*':ti,ab | 254,485 |
| #6 | #4 OR #5 | 351,657 |
| #7 | exercise'/exp OR 'motor activity'/exp OR 'sedentary behavior'/exp OR 'walking'/exp | 1,425,905 |
| #8 | physical activity':ti,ab OR exercise*:ti,ab OR walking:ti,ab OR 'daily steps':ti,ab OR 'step count*':ti,ab OR mvpa:ti,ab OR sedentary:ti,ab OR 'sitting time':ti,ab OR acceleromet*:ti,ab OR 'active lifestyle':ti,ab | 926,668 |
| #9 | #7 OR #8 | 1,825,629 |
| #10 | randomized controlled trial'/exp OR 'randomization'/exp | 1,227,197 |
| #11 | randomized:ti,ab OR randomised:ti,ab OR rct:ti,ab OR 'random allocation':ti,ab OR 'clinical trial*':ti,ab | 2,208,265 |
| #12 | #10 OR #11 | 2,507,348 |
| #13 | #3 AND #6 AND #9 AND #12 | 4,256 |

**Database: The Cochrane Library (via Wiley)**

**Date of Search: February 20, 2026**

| **Step** | **Search Terms** | **Results**  **(Trials)** |
| --- | --- | --- |
| #1 | [Mesh descriptor: [Aged] explode all trees] | 289,571 |
| #2 | older adult* OR elderly OR senior* OR geriatric* OR aging OR ageing | 141,998 |
| #3 | #1 OR #2 | 388,404 |
| #4 | [Mesh descriptor: [Telemedicine] explode all trees] | 5,984 |
| #5 | [Mesh descriptor: [Mobile Applications] explode all trees] | 2,816 |
| #6 | [Mesh descriptor: [Wearable Electronic Devices] explode all trees] | 1,214 |
| #7 | [Mesh descriptor: [Smartphone] explode all trees] | 1,386 |
| #8 | [Mesh descriptor: [Fitness Trackers] explode all trees] | 273 |
| #9 | digital health OR mhealth OR ehealth OR telehealth OR (mobile NEXT app*) OR wearable* OR smartphone* OR pedometer* OR (activity NEXT tracker*) OR (internet NEXT based) OR (web NEXT based) OR (online NEXT intervention*) OR (text NEXT messag*) | 60,852 |
| #10 | #4 OR #5 OR #6 OR #7 OR #8 OR #9 | 63,792 |
| #11 | [Mesh descriptor: [Exercise] explode all trees] | 42,079 |
| #12 | [Mesh descriptor: [Motor Activity] explode all trees] | 45,976 |
| #13 | [Mesh descriptor: [Sedentary Behavior] explode all trees] | 2,081 |
| #14 | [Mesh descriptor: [Walking] explode all trees] | 8,697 |
| #15 | physical activity OR exercise* OR walking OR (daily NEXT steps) OR (step NEXT count*) OR mvpa OR sedentary OR (sitting NEXT time) OR acceleromet* OR (active NEXT lifestyle) | 226,712 |
| #16 | #11 OR #12 OR #13 OR #14 OR #15 | 230,639 |
| #17 | #3 AND #10 AND #16 | 4,199 |

**Database: CINAHL (via EBSCOhost)**

**Date of Search: February 20, 2026**

| **Step** | **Search Terms** | **Results** |
| --- | --- | --- |
| #1 | (MH "Aged+") OR (MH "Aged, 80 and Over") | 1,022,406 |
| #2 | XB (older adult* OR elderly OR senior* OR geriatric* OR aging OR ageing) | 314,432 |
| #3 | #1 OR #2 | 1,147,803 |
| #4 | (MH "Telemedicine+") OR (MH "Mobile Applications") OR (MH "Wearable Technology") | 48,248 |
| #5 | XB (digital health OR mHealth OR eHealth OR telehealth OR mobile app* OR wearable* OR smartphone* OR pedometer* OR activity tracker* OR internet-based OR web-based OR online intervention* OR text messag*) | 75,726 |
| #6 | #4 OR #5 | 110,259 |
| #7 | (MH "Physical Activity+") OR (MH "Sedentary Behavior") OR (MH "Walking") OR (MH "Exercise+") | 205,696 |
| #8 | XB (physical activity OR exercise* OR walking OR daily steps OR step count* OR MVPA OR sedentary OR sitting time OR acceleromet* OR active lifestyle) | 267,149 |
| #9 | #7 OR #8 | 351,573 |
| #10 | (MH "Randomized Controlled Trials") | 160,502 |
| #11 | XB (randomized OR randomised OR RCT OR clinical trial*) | 442,728 |
| #12 | #10 OR #11 | 485,061 |
| #13 | #3 AND #6 AND #9 AND #12 | 944 |

**Deduplication Process:** All retrieved records were imported into EndNote. Automatic deduplication was performed using the software’s “Find Duplicates” function, followed by a manual review of the remaining records by two independent reviewers to ensure all duplicate entries were removed before screening.
