# Supplementary material for: Effects of Multicomponent Digital Health Interventions on Multidimensional Physical Activity in Older Adults: Systematic Review, Meta-Analysis, and Meta-Regression of Randomized Controlled Trials
Source: J Med Internet Res. 2026 May 29;28:e91338. doi: 10.2196/91338 (PMC13221160; doi:10.2196/91338)
Supplement: Multimedia Appendix 2 — Evidence and robustness analyses, including leave-one-out sensitivity analysis, HKSJ random-effects forest plots with 95% PIs for primary and subgroup outcomes, and meta-regression influence diagnostics. HKSJ: Hartung-Knapp-Sidik-Jonkman; PI: prediction interval. [file jmir-v28-e91338-s002.docx]

**Table S1.** Leave-one-out sensitivity analysis to assess the robustness of the pooled effect of multi-component digital health interventions on daily steps in older adults.

| **Omitting Study** | **MD** | **95% CI** | **I^2^** |
| --- | --- | --- | --- |
| All studies | 822.8 | [198.3, 1447.3] | 67.5% |
| Ashe et al., 2015 | 779.6 | [124.3, 1434.9] | 68.6% |
| Bickmore et al., 2013 | 870.3 | [183.5, 1557.2] | 69.9% |
| Brickwood et al., 2021 | 817.8 | [133.7, 1501.9] | 69.1% |
| Cai et al., 2022 | 877.4 | [189.3, 1565.5] | 69.9% |
| Kawaguchi et al., 2024 | 938.3 | [313.9, 1562.8] | 63.3% |
| Kwan et al., 2020 | 707.3 | [146.8, 1267.8] | 66.4% |
| Liu et al., 2021 | 900.2 | [224.3, 1576.1] | 67.6% |
| Oliveira et al., 2019 | 883.6 | [200.9, 1566.4] | 69.9% |
| Oliveira et al., 2024 | 833.5 | [146.4, 1520.6] | 69.5% |
| Recio-R et al., 2022 | 901.4 | [252.3, 1550.5] | 69.2% |
| Roberts et al., 2019 | 750.5 | [100.8, 1400.1] | 66.6% |
| Rosenberg et al., 2020 | 873.7 | [195.6, 1551.8] | 69.9% |
| Yates et al., 2009 | 730.8 | [83.4, 1378.3] | 62.2% |
| Zhou et al., 2021 | 677.8 | [89.3, 1266.4] | 60.1% |


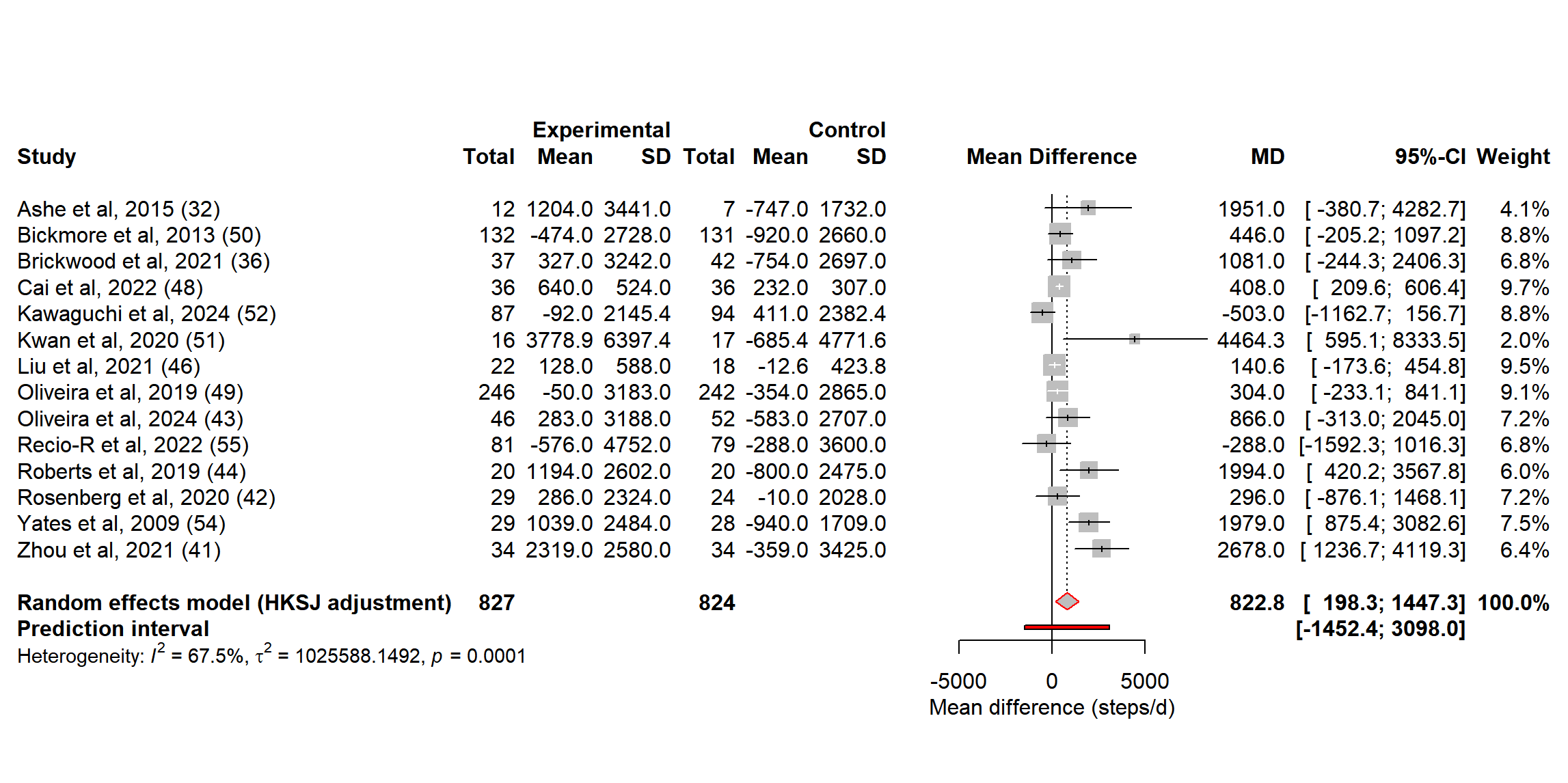


**Figure S1.** Meta-analysis forest plot of randomized controlled trials evaluating the effects of multicomponent digital health interventions on daily steps (steps/d) in older adults based on the HKSJ random-effects model and 95% PIs. HKSJ: Hartung-Knapp-Sidik-Jonkman; MD: mean difference; PI: prediction interval. [32,36,41-44,46,48-52,54,55].


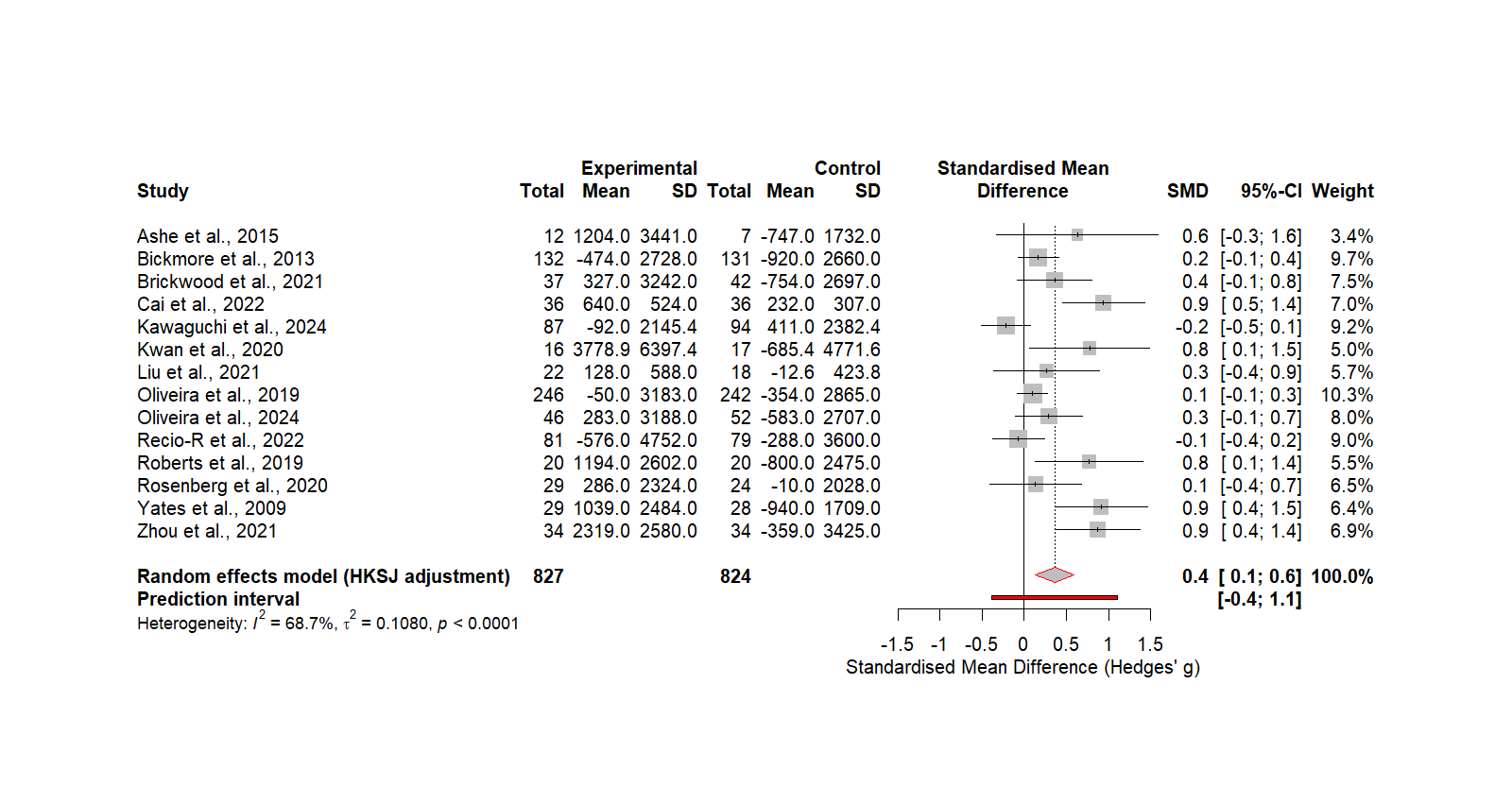


**Figure S2.** Forest plot using the HKSJ random-effects model and 95% prediction interval with standardized mean difference (SMD, Hedges’ g) as the effect metric to evaluate the robustness of multi-component digital health interventions on daily steps in older adults.


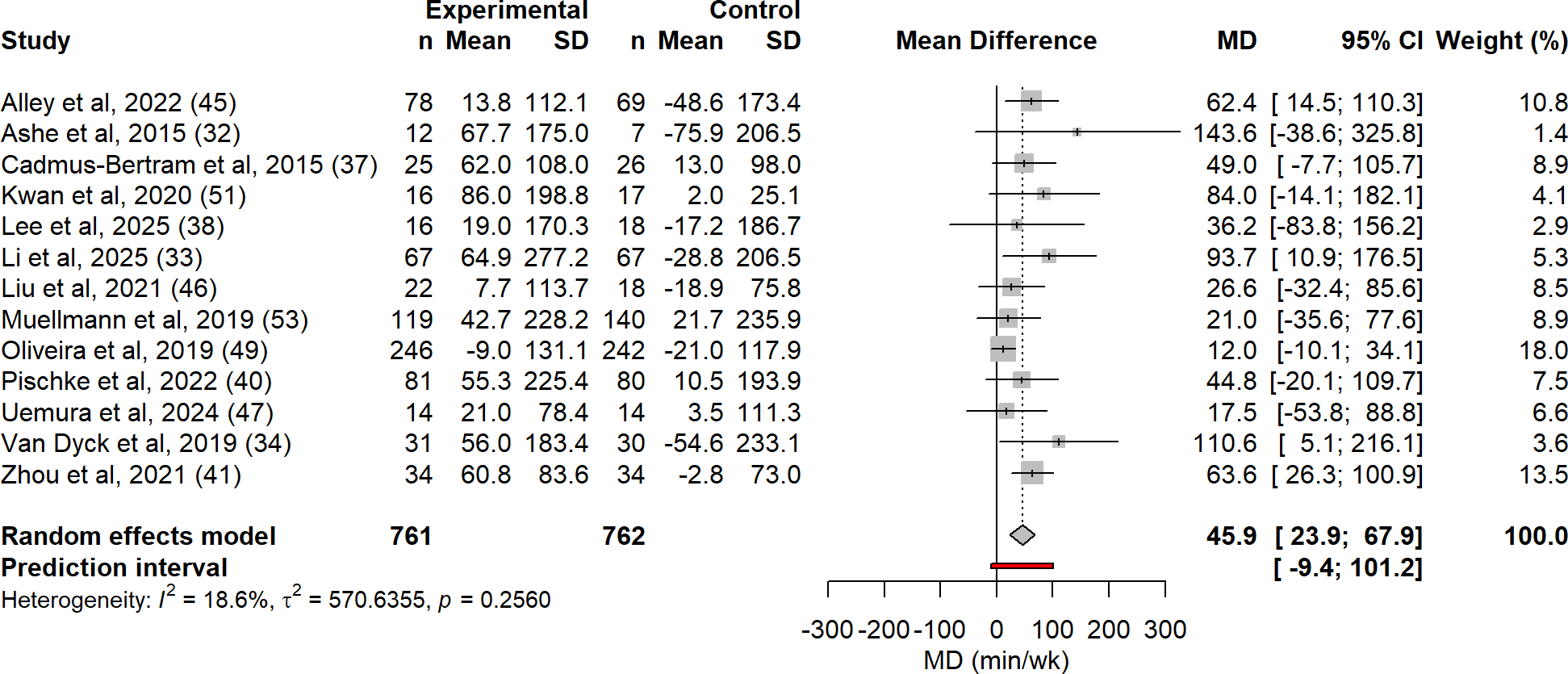
**Figure S3.** Meta-analysis forest plot of randomized controlled trials evaluating the effects of multicomponent digital health interventions on weekly moderate-to-vigorous physical activity time (min/wk) in older adults based on the HKSJ random-effects model and 95% PIs. HKSJ: Hartung-Knapp-Sidik-Jonkman; MD: mean difference; PI: prediction interval. [32-34,37,38,40,41,45-47,49,51,53].


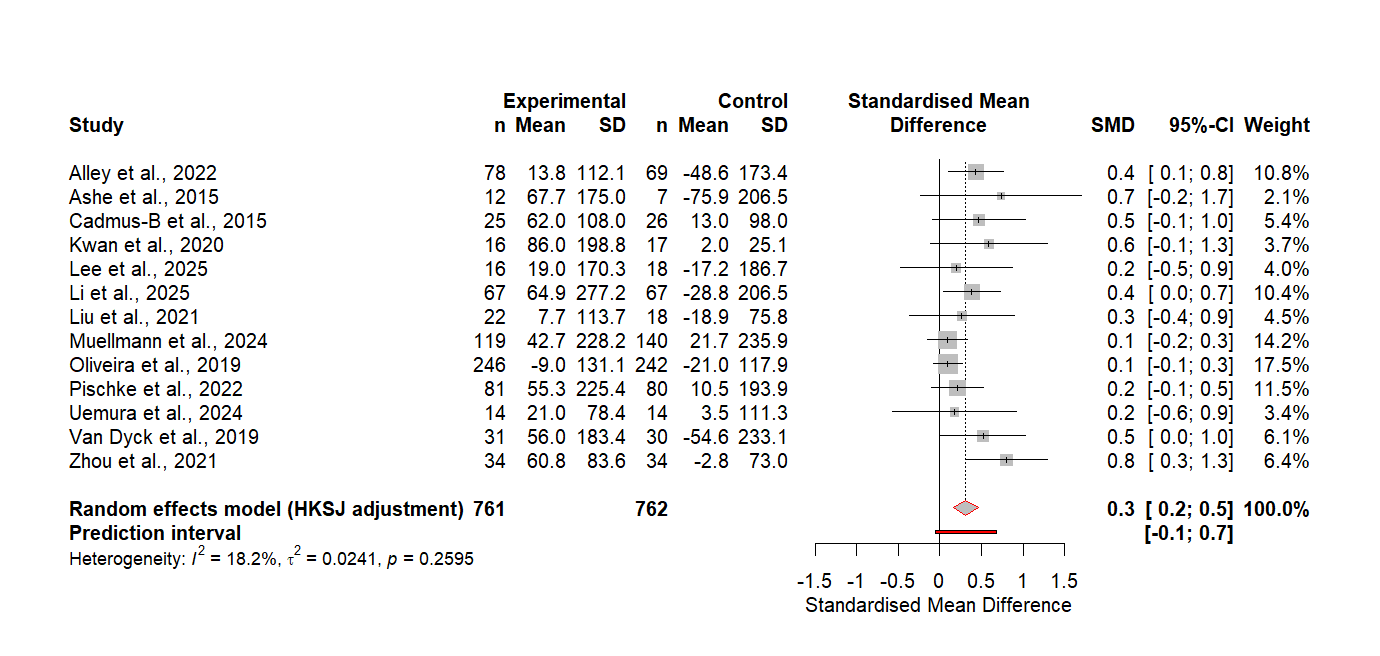


**Figure S4.** Forest plot using the HKSJ random-effects model and 95% prediction interval with standardized mean difference (SMD, Hedges’ g) as the effect metric to evaluate the robustness of multi-component digital health interventions on weekly MVPA time in older adults.


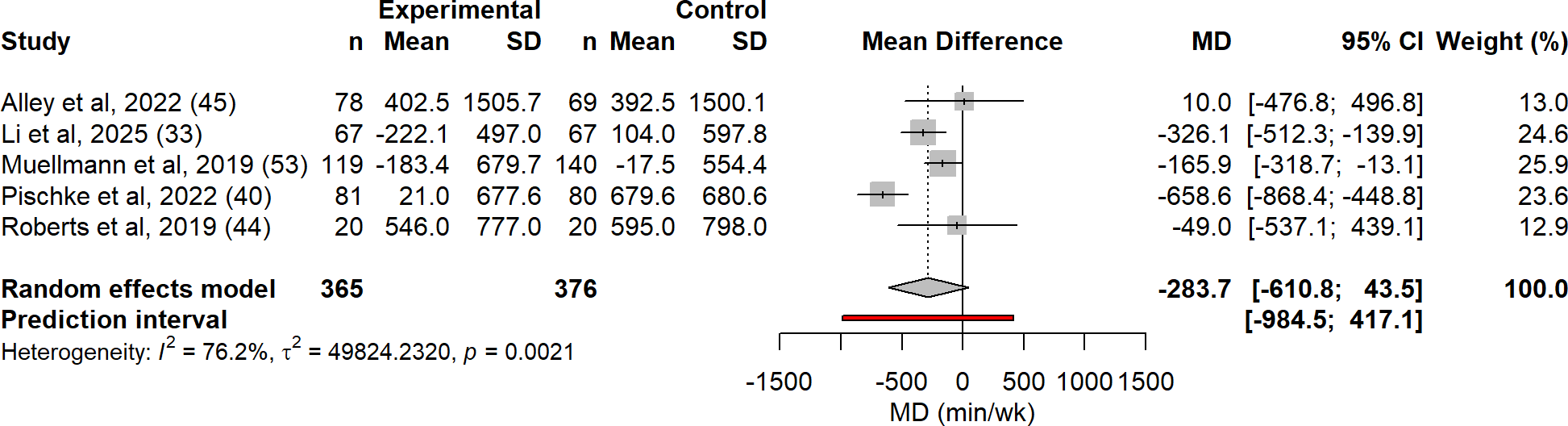
**Figure S5.** Meta-analysis forest plot of randomized controlled trials evaluating the effects of multicomponent digital health interventions on weekly sedentary time (min/wk) in older adults based on the HKSJ random-effects model and 95% PIs. HKSJ: Hartung-Knapp-Sidik-Jonkman; MD: mean difference; PI: prediction interval [33,40,44,45,53].
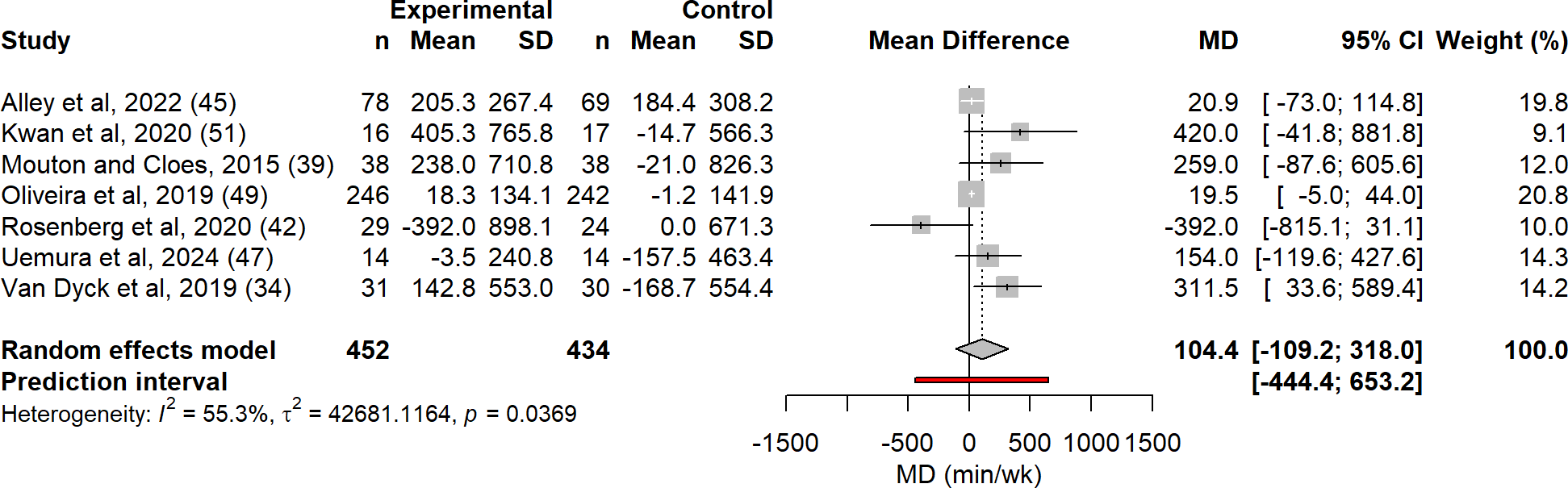
**Figure S6**. Meta-analysis forest plot of randomized controlled trials evaluating the effects of multicomponent digital health interventions on weekly total physical activity time (min/wk) in older adults based on the HKSJ random-effects model and 95% PIs. HKSJ: Hartung-Knapp-Sidik-Jonkman; MD: mean difference; PI: prediction interval [34,39,42,45,47,49,51].


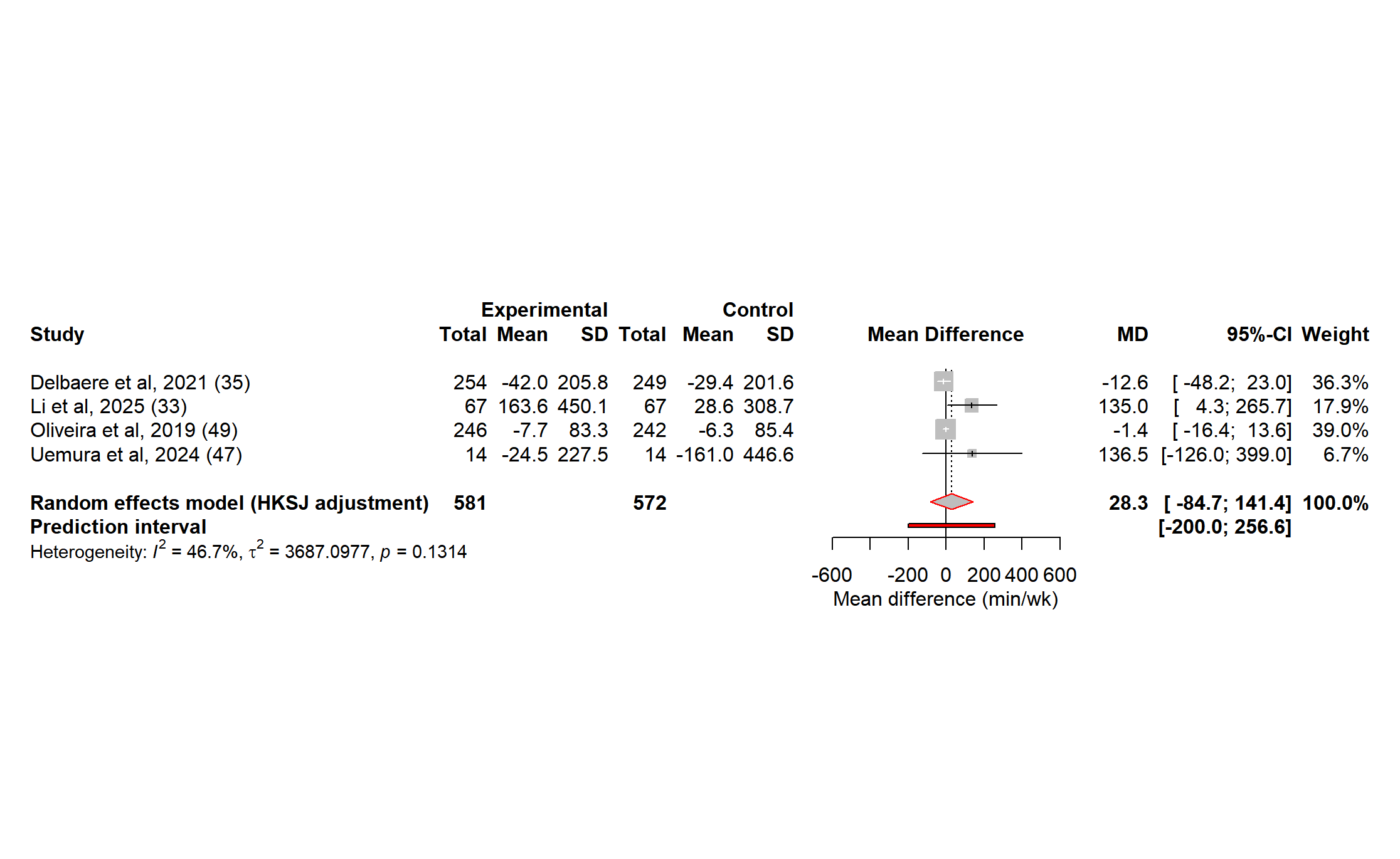


**Figure S7.** Meta-analysis forest plot of randomized controlled trials evaluating the effects of multicomponent digital health interventions on light physical activity time (min/wk) in older adults based on the HKSJ random-effects model and 95% PIs. HKSJ: Hartung-Knapp-Sidik-Jonkman; MD: mean difference; PI: prediction interval [33,47,49,56].


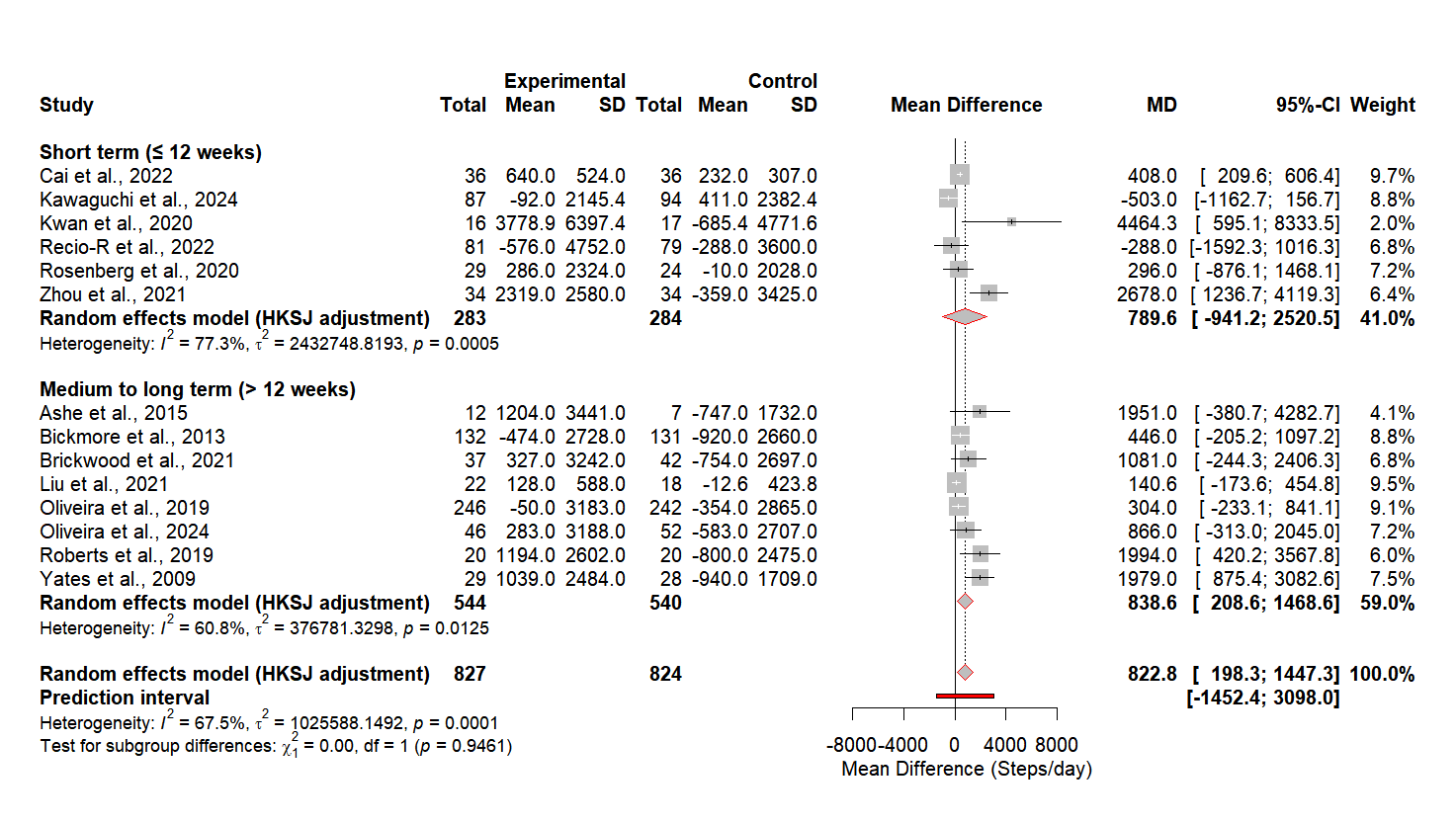


**Figure S8.** Forest plot using the HKSJ random-effects model and 95% prediction interval to evaluate the effects of multi-component digital health interventions on daily steps in older adults categorized by intervention duration.


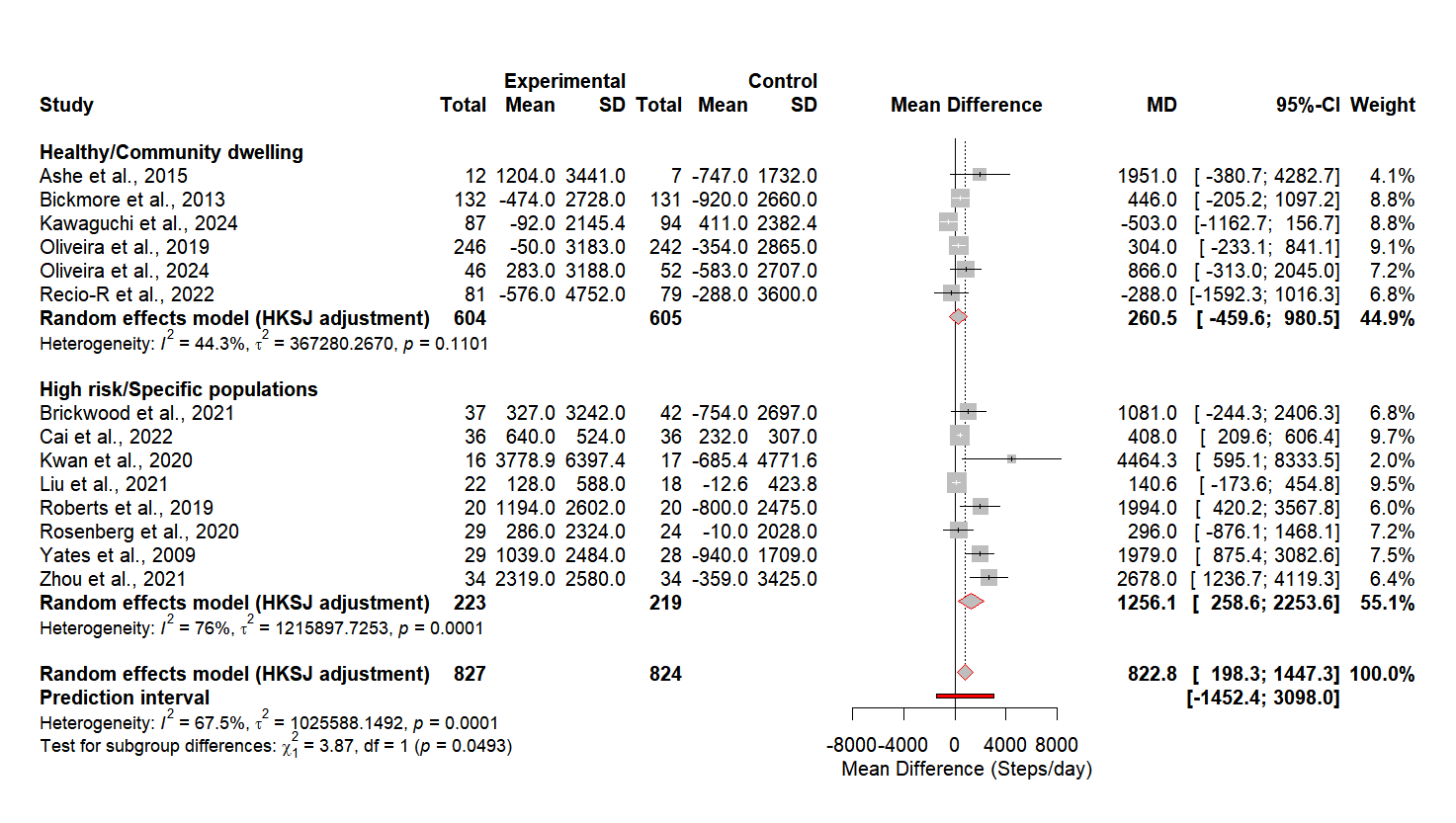


**Figure S9.** Forest plot using the HKSJ random-effects model and 95% prediction interval to evaluate the effects of multi-component digital health interventions on daily steps in older adults categorized by population type.


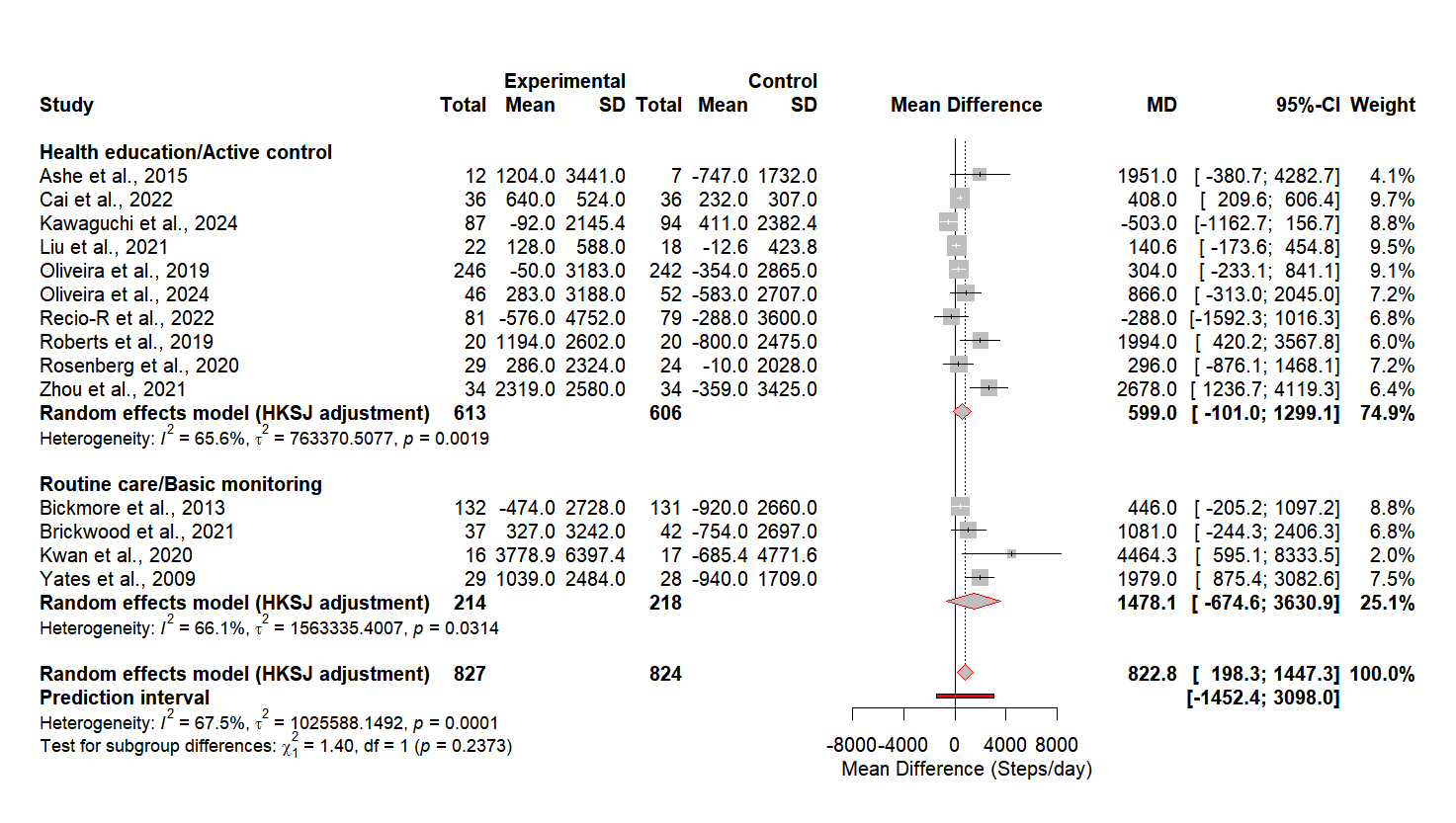


**Figure S10.** Forest plot using the HKSJ random-effects model and 95% prediction interval to evaluate the effects of multi-component digital health interventions on daily steps in older adults categorized by control group settings.


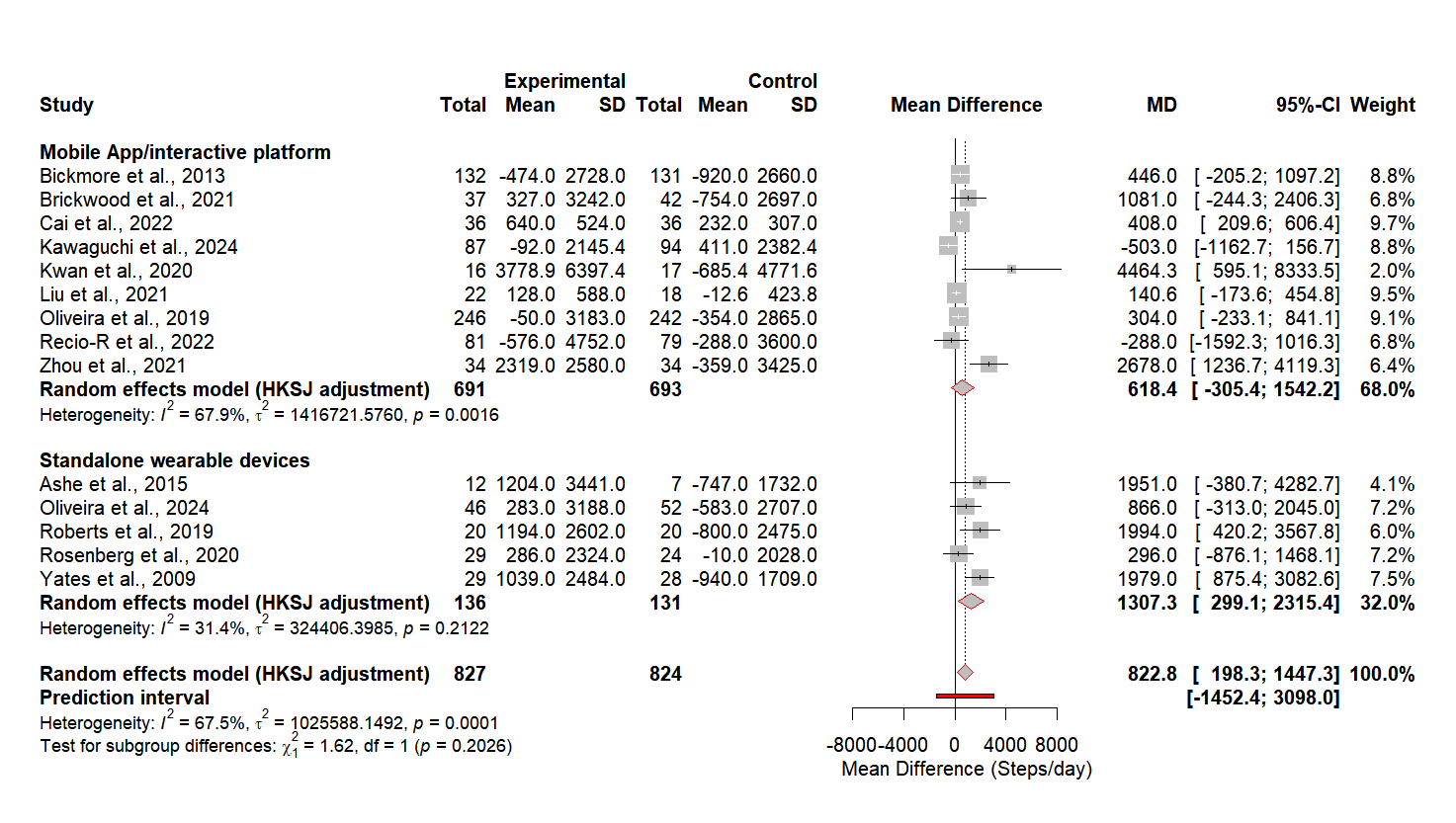


**Figure S11.** Forest plot using the HKSJ random-effects model and 95% prediction interval to evaluate the effects of multi-component digital health interventions on daily steps in older adults categorized by technology carrier.


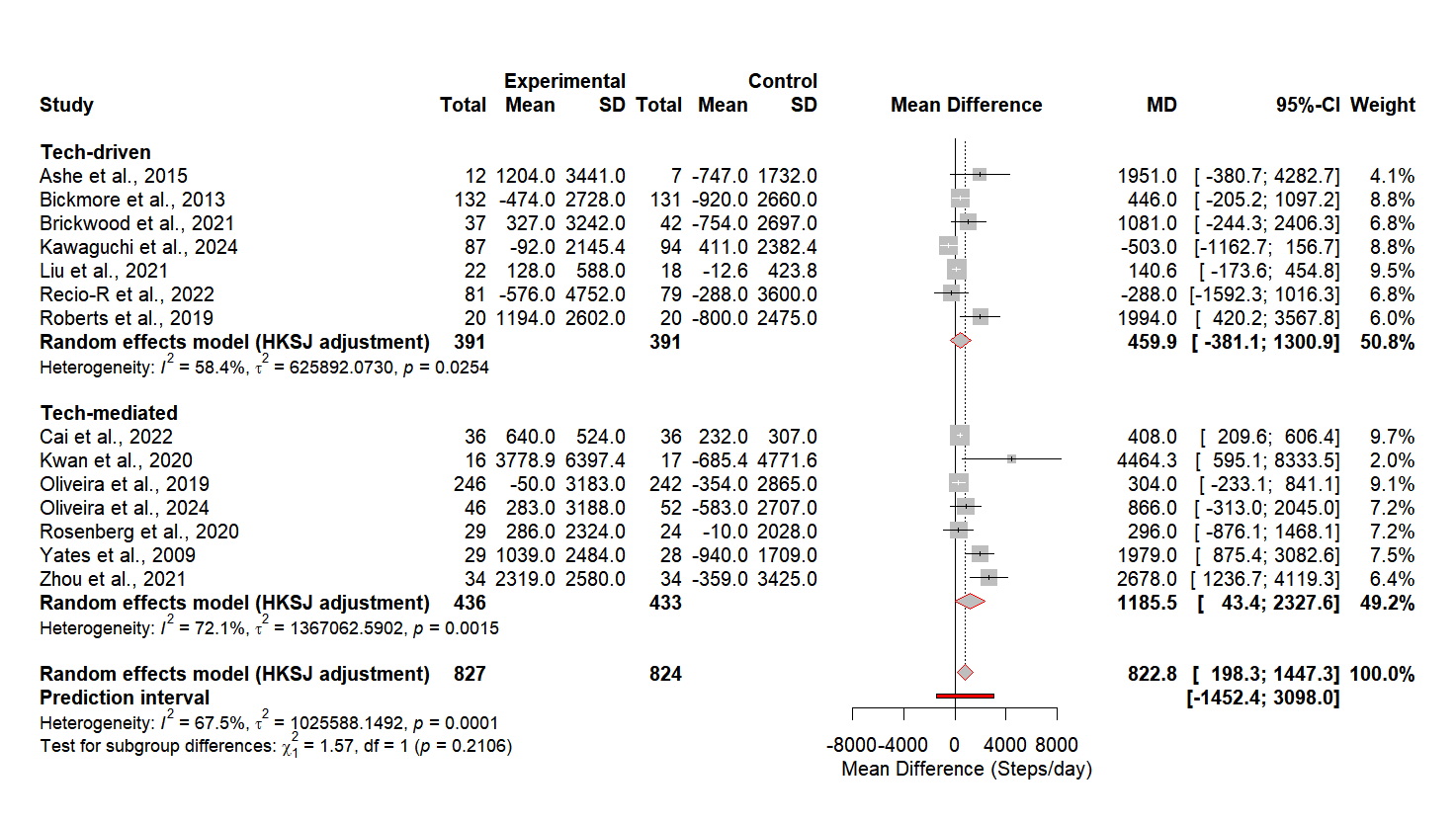


**Figure S12.** Forest plot using the HKSJ random-effects model and 95% prediction interval to evaluate the effects of multi-component digital health interventions on daily steps in older adults categorized by delivery agency.


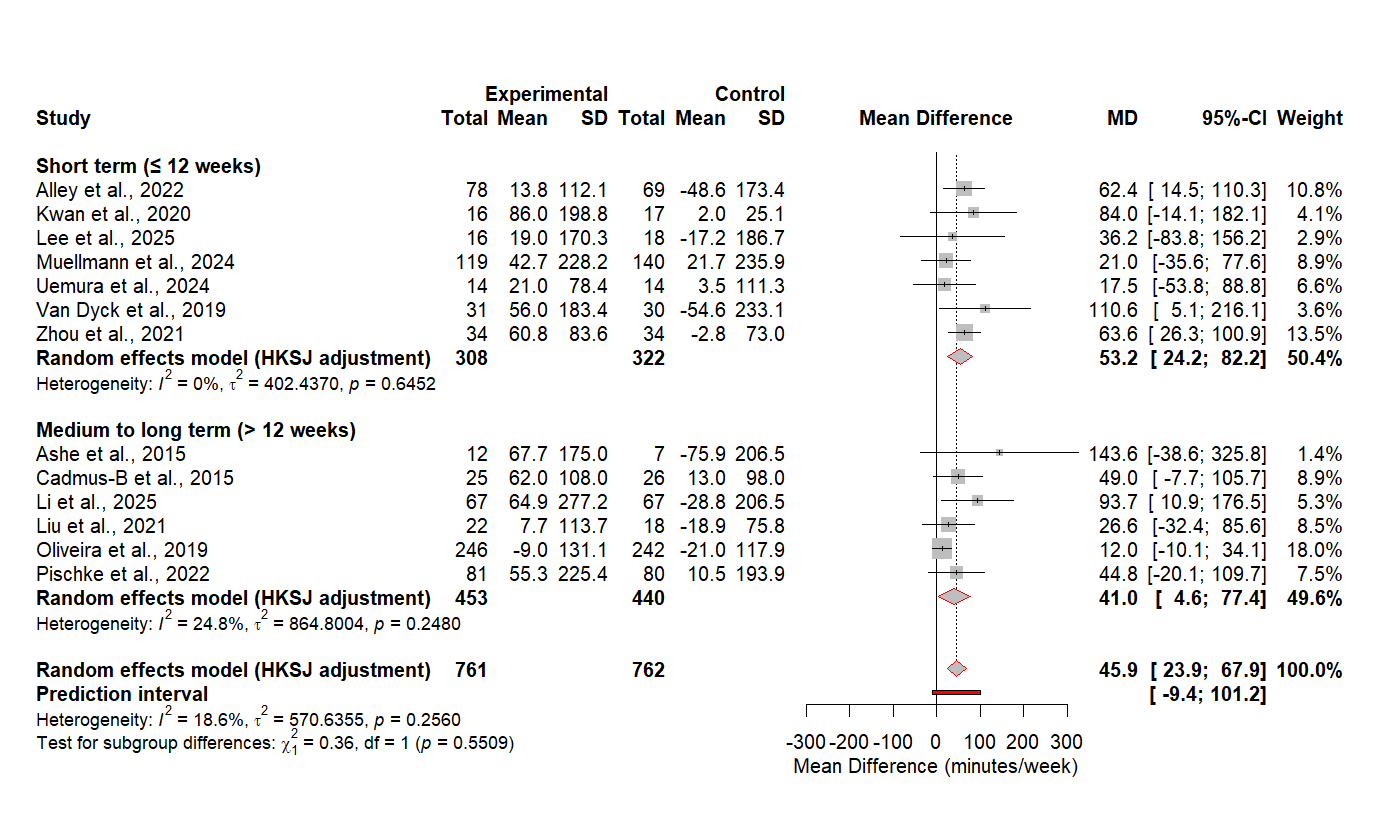


**Figure S13.** Forest plot using the HKSJ random-effects model and 95% prediction interval to evaluate the effects of multi-component digital health interventions on weekly MVPA time in older adults categorized by intervention duration.


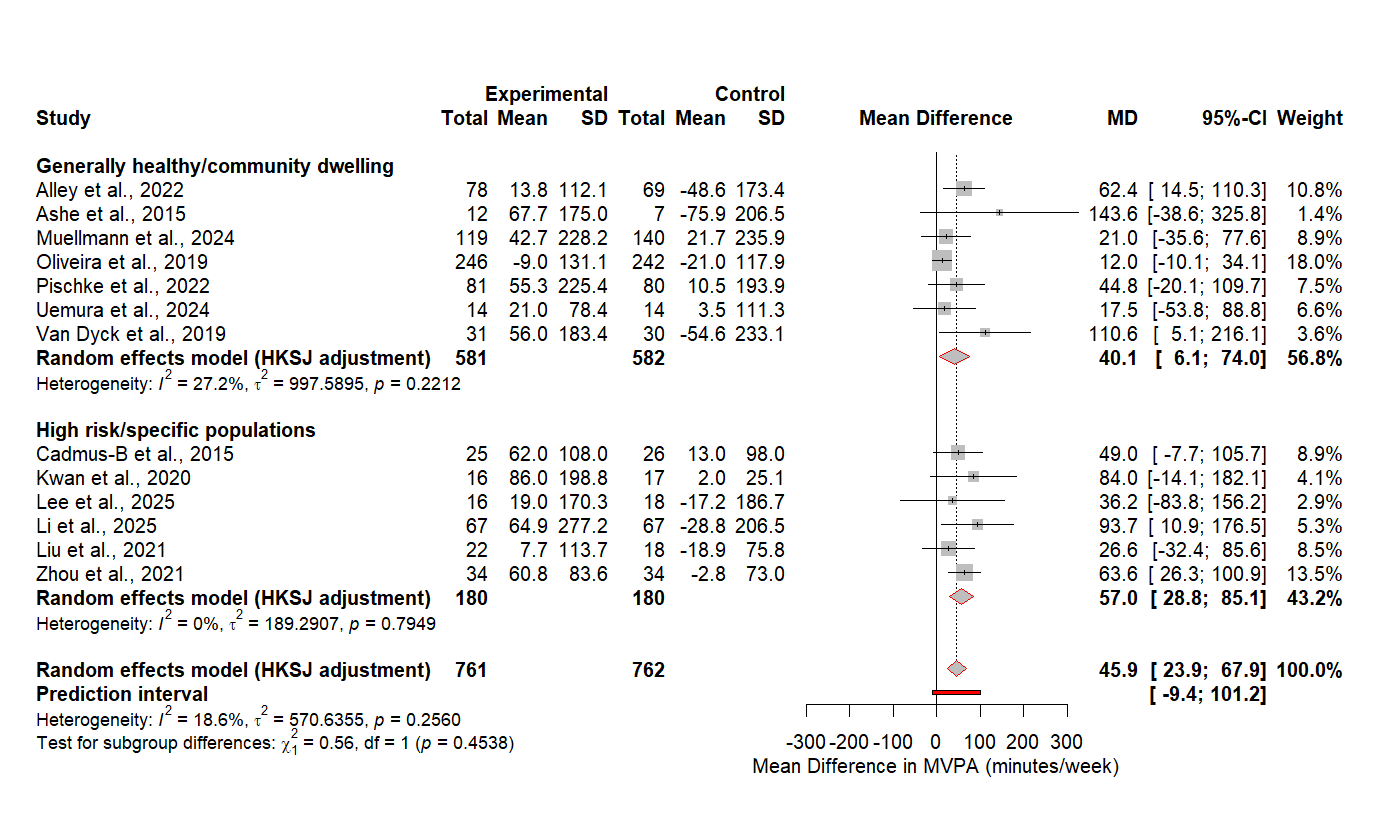


**Figure S14.** Forest plot using the HKSJ random-effects model and 95% prediction interval to evaluate the effects of multi-component digital health interventions on weekly MVPA time in older adults categorized by population type.


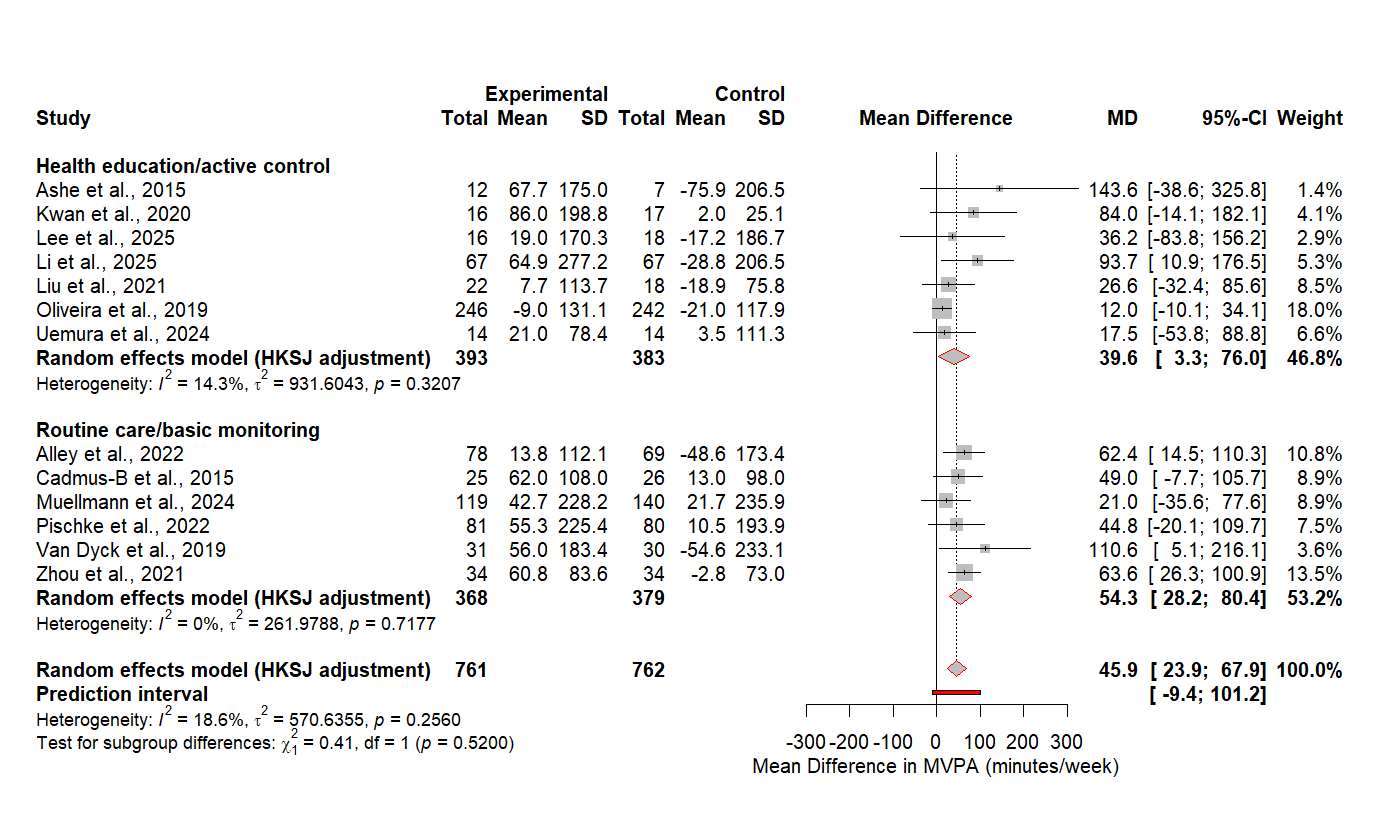


**Figure S15.** Forest plot using the HKSJ random-effects model and 95% prediction interval to evaluate the effects of multi-component digital health interventions on weekly MVPA time in older adults categorized by control group settings.


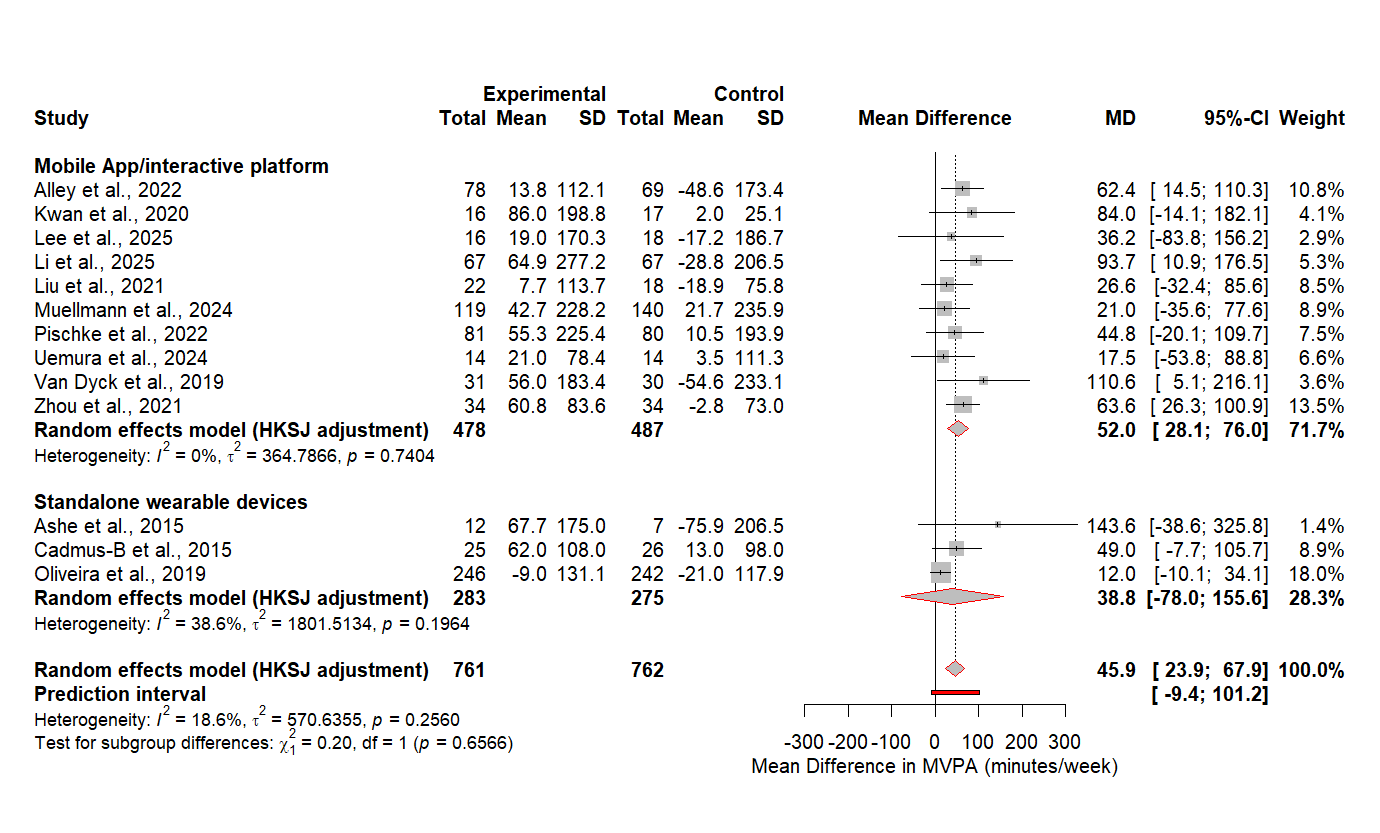


**Figure S16.** Forest plot using the HKSJ random-effects model and 95% prediction interval to evaluate the effects of multi-component digital health interventions on weekly MVPA time in older adults categorized by technology carrier.


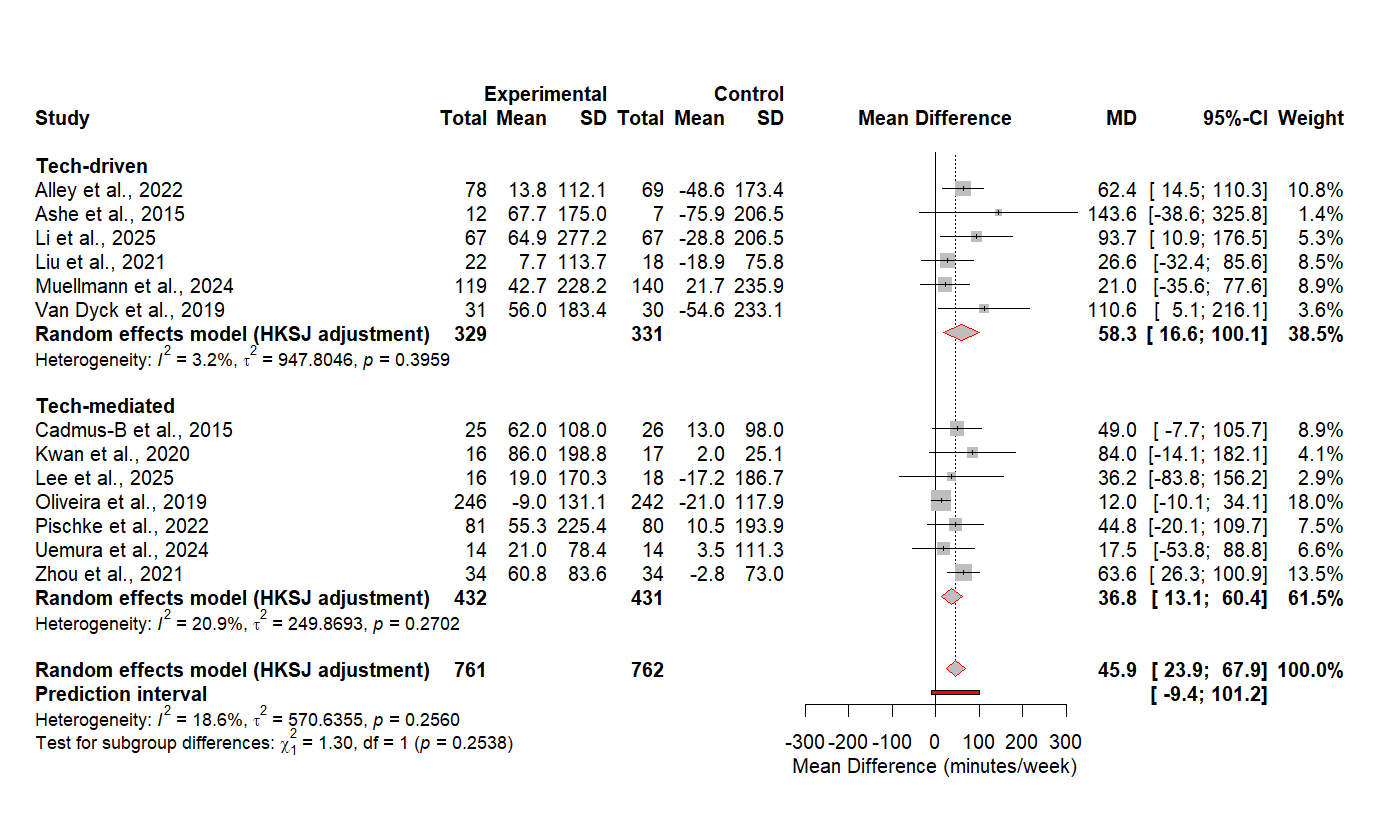


**Figure S17.** Forest plot using the HKSJ random-effects model and 95% prediction interval to evaluate the effects of multi-component digital health interventions on weekly MVPA time in older adults categorized by delivery agency.


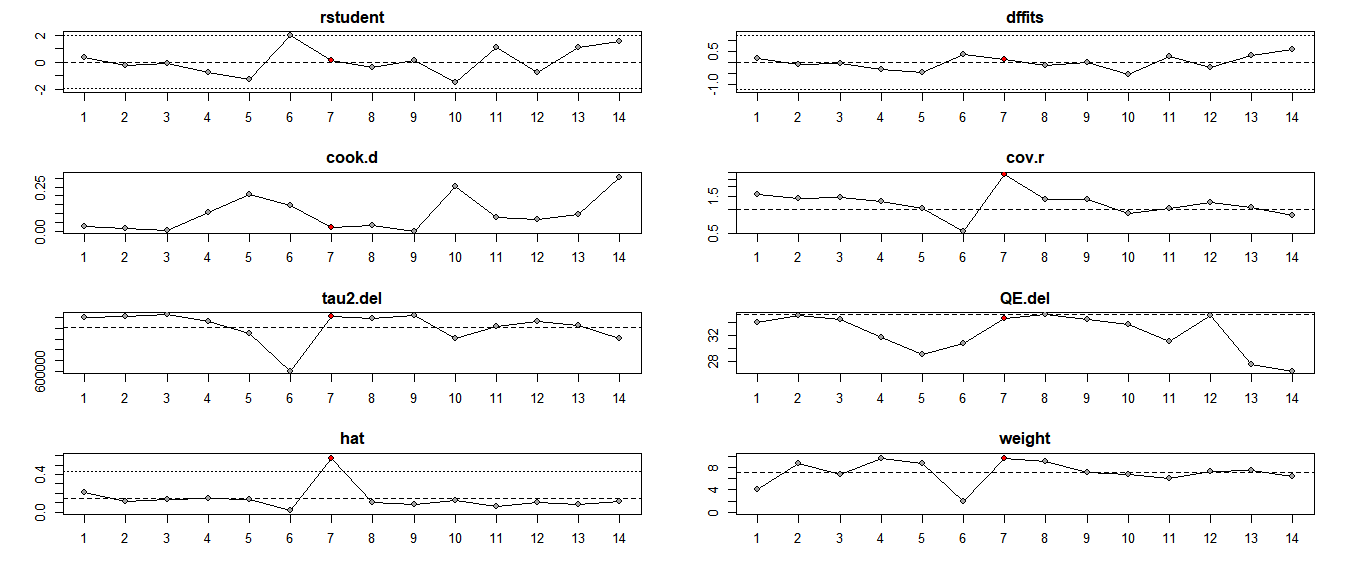


**Figure S18.** Influence diagnostics of the meta-regression model for daily steps in older adults using age as a moderator.

Note: This plot displays multi-dimensional diagnostic metrics (e.g., studentized residuals, Cook’s distance, covariance ratio, and hat values) to identify potential outliers. All studies exhibited a Cook’s distance < 0.31, indicating high model stability.


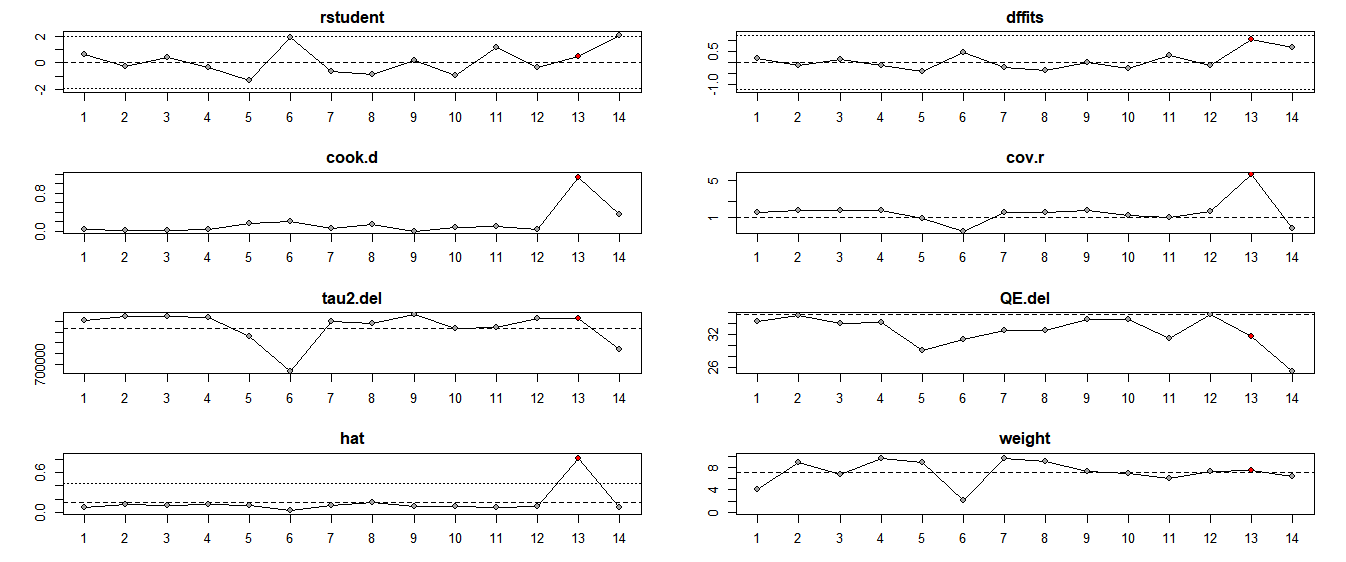


**Figure S19.** Influence diagnostics of the meta-regression model for daily steps in older adults using intervention duration as a moderator.

Note: The diagnostic indicators are uniformly distributed, with no influential studies identified as exerting excessive impact on the parameter estimates.


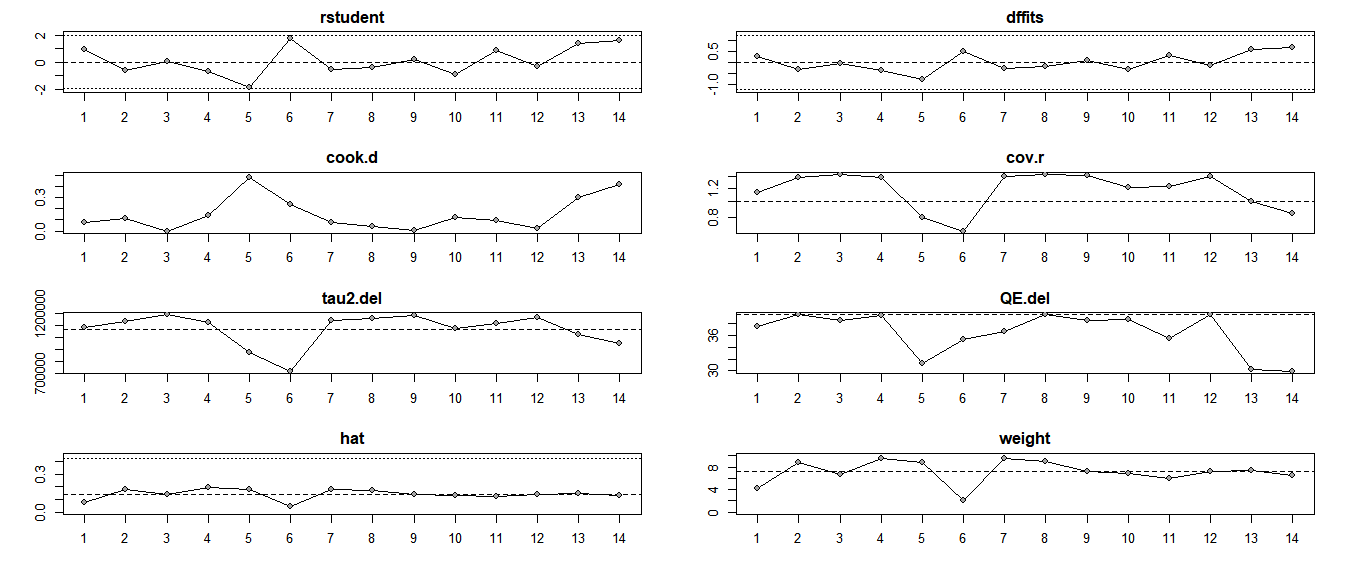

**Figure S20.** Influence diagnostics of the meta-regression model for daily steps in older adults using prompting frequency as a moderator.

Note: All study data points fall within reasonable ranges for Cook’s distance and hat values, confirming the robustness of the regression results.


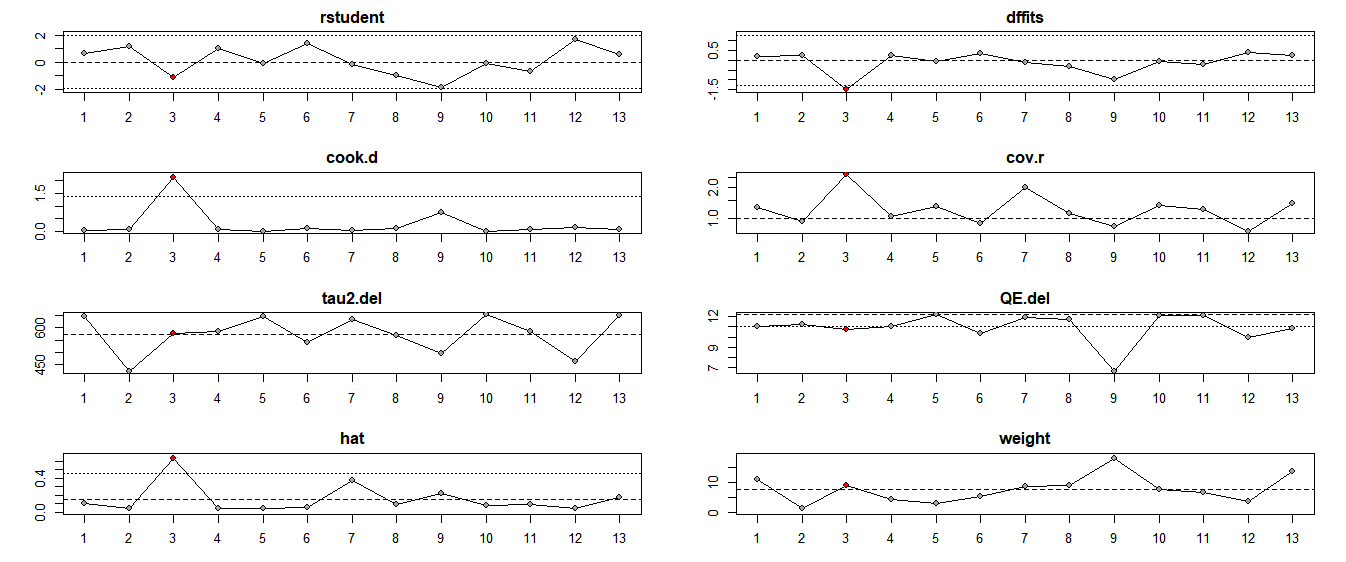

**Figure S21.** Influence diagnostics of the meta-regression model for MVPA in older adults using age as a moderator.

Note: Red asterisks (*) identify Study No. 3 as having a high Cook’s distance (Cook’s D = 2.14) and hat value, indicating its potential influence on the regression coefficients.
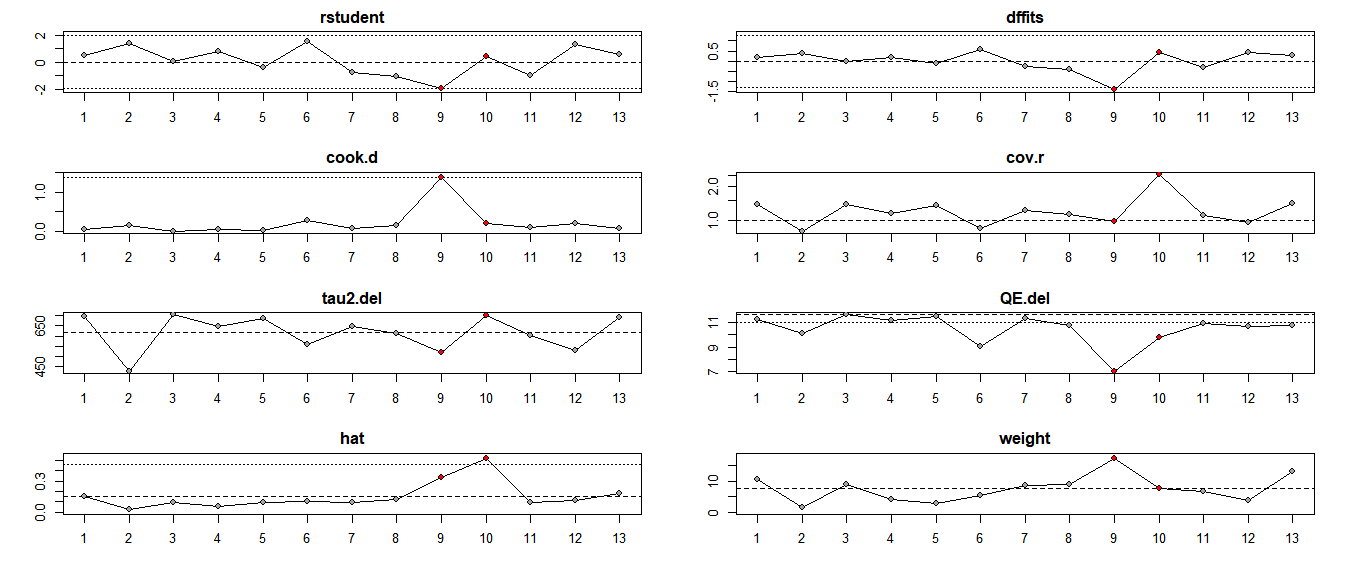

**Figure S22.** Influence diagnostics of the meta-regression model for MVPA in older adults using intervention duration as a moderator.

Note: Results identify Study No. 9 (Cook's D = 1.39) and Study No. 10 as influential cases, highlighted in red within the diagnostic plots.


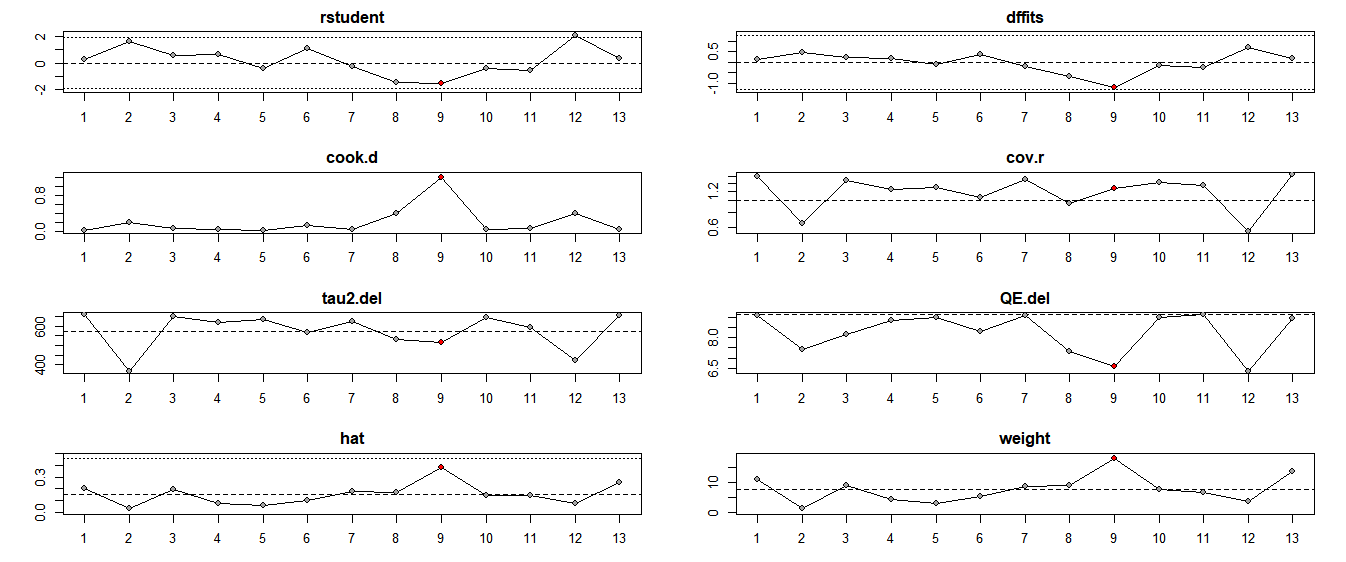

**Figure S23.** Influence diagnostics of the meta-regression model for MVPA in older adults using prompting frequency as a moderator.

Note: Red asterisks (*) mark Study No. 9 as a potential influential case (Cook’s D = 1.19), exhibiting higher values in covariance ratio and Cook's distance.
