# Supplementary material for: Effects of Multicomponent Digital Health Interventions on Multidimensional Physical Activity in Older Adults: Systematic Review, Meta-Analysis, and Meta-Regression of Randomized Controlled Trials
Source: J Med Internet Res. 2026 May 29;28:e91338. doi: 10.2196/91338 (PMC13221160; doi:10.2196/91338)
Supplement: Checklist 3 — PRISMA-S checklist which details the reporting of literature search strategies across 16 items, specifically mapping information sources, search strings, and deduplication processes to their respective locations in the manuscript and Multimedia Appendix 1. [file jmir-v28-e91338-s005.pdf]

PRISMA-S Checklist

Section/topic	#	Checklist item	Location(s) Reported
**INFORMATION SOURCES AND METHODS**			
Database name	1	Name each individual database searched, stating the platform for each.	Page 4, lines 161–163
Multi-database searching	2	If databases were searched simultaneously on a single platform, state the name of the platform, listing all of the databases searched.	Page 4, lines 161–163
Study registries	3	List any study registries searched.	Page 4, lines 161–163
Online resources and browsing	4	Describe any online or print source purposefully searched or browsed (e.g., tables of contents, print conference proceedings, web sites), and how this was done.	Page 4, lines 185-187
Citation searching	5	Indicate whether cited references or citing references were examined, and describe any methods used for locating cited/citing references (e.g., browsing reference lists, using a citation index, setting up email alerts for references citing included studies).	Page 4, lines 178-179
Contacts	6	Indicate whether additional studies or data were sought by contacting authors, experts, manufacturers, or others.	Page 4, lines 184-185
Other methods	7	Describe any additional information sources or search methods used.	Page 4, lines 184-185
SEARCH STRATEGIES			
Full search strategies	8	Include the search strategies for each database and information source, copied and pasted exactly as run.	Page 4, lines 171–182 and Supplementary Text 1
Limits and restrictions	9	Specify that no limits were used, or describe any limits or restrictions applied to a search (e.g., date or time period, language, study design) and provide justification for their use.	Page 4, lines 167–188
Search filters	10	Indicate whether published search filters were used (as originally designed or modified), and if so, cite the filter(s) used.	Page 4, line 185-186

Prior work	11	Indicate when search strategies from other literature reviews were adapted or reused for a substantive part or all of the search, citing the previous review(s).	Page 4, lines 187-189
Updates	12	Report the methods used to update the search(es) (e.g., rerunning searches, email alerts).	Page 4, line 161
Dates of searches	13	For each search strategy, provide the date when the last search occurred.	Page 4, line 161
PEER REVIEW			
Peer review	14	Describe any search peer review process.	Page 4, lines 183-185
MANAGING RECORDS			
Total Records	15	Document the total number of records identified from each database and other information sources.	Page 7, lines 301-304
Deduplication	16	Describe the processes and any software used to deduplicate records from multiple database searches and other information sources.	Page 7, lines 304-305

PRISMA-S: An Extension to the PRISMA Statement for Reporting Literature Searches in Systematic Reviews
Rethlefsen ML, Kirtley S, Waffenschmidt S, Ayala AP, Moher D, Page MJ, Koffel JB, PRISMA-S Group.
Last updated February 27, 2020.
